# North Atlantic influence reconciling model-observation discrepancy in the tropical Pacific warming pattern

**DOI:** 10.1038/s41467-026-73763-0

**Published:** 2026-05-28

**Authors:** Yueh-Chi Lin, Masahiro Watanabe

**Affiliations:** Atmosphere and Ocean Research Institute, University of Tokyo, Kashiwa, Japan

**Keywords:** Attribution, Climate and Earth system modelling

## Abstract

Over the past four decades, zonal contrast in the tropical Pacific sea surface temperature (SST) has strengthened in observations but weakened in majority of climate model simulations. This model–observation discrepancy cannot be explained by internal mode of interdecadal climate variability in the Pacific alone, and the source of possible model errors remains unclear. Here, using observations and a large ensemble of historical simulations by a climate model, we identified that the simulated SST pattern associated with the Atlantic Multidecadal Variability (AMV) is biased in the tropical Pacific despite the time evolution of the AMV being reproduced well. Observations suggest that the positive AMV acts to increase the Pacific zonal SST contrast whereas this teleconnection process falsely weakens it in the model, which is a common feature in other climate models, and correcting the AMV-related SST pattern, which is likely an externally forced response, partly reconciles the model-observation discrepancy.

## Abstract (web summary)

This study shows that climate models underestimate the strengthening of the Pacific east–west temperature contrast observed in recent decades. The mismatch arises from poorly represented **North Atlantic warming influences on the tropical Pacific in the models**, but correcting spatial biases improves agreement with observations.

## Introduction

Sea surface temperature (SST) in the tropical Pacific is far from uniform, higher in the west and lower in the east, driving atmospheric Walker circulation and controlling many features of the global climate system. Slight change in the SST pattern has a great impact on world’s weather such as tropical cyclones^1^. Future climate projections suggest that the zonal SST contrast in the tropical Pacific will weaken with increasing global warming level^2^, which is supposed to emerge at present with increasing global surface temperature of about 1.1 K since the preindustrial era^3^.

However, observational data show that the zonal SST contrast has intensified over the past four decades, in contrast to the future projections by Coupled Model Intercomparison Phase 6 (CMIP6) climate models. Because the Pacific SST trends at multidecadal time scales are influenced by a phase of the interdecadal Pacific oscillation (IPO), an internal mode of climate variability^4^ and sharing substantial spatial-temporal features with the Pacific decadal oscillation (PDO)^5^ and the tropical Pacific decadal variability^6^, the above strengthening of the zonal SST contrast was initially thought of arising from the negative IPO lasting after the late 1970s^7,8^. A thorough analysis of CMIP6 large-ensemble historical simulations has shown that the models overall failed to reproduce the observed SST contrast change as they rather simulated the SST contrast to weaken as in the future projections^9^. Although the estimated tropical Pacific SST trend depends on the choice of period^10^ because low-frequency internal variability may affect the trend estimated over a given interval, a discrepancy still exists between observations and climate models regarding the past change in the tropical Pacific zonal SST contrast, raising a critical concern about reliability of the future climate projections.

Two major scenarios that explain the model-observation discrepancy have been proposed^11–13^. First, models may underestimate the amplitude of the IPO, narrowing their ensemble spread so that the observed trend is not captured. This possibility is not likely as some models do show a considerable large ensemble spread in the zonal SST contrast trend, which yet is systematically shifted from the observations^9^. Second, models may misrepresent or lack part of externally forced response. This models’ inability could come from biases in the tropical Pacific mean state^14,15^, weak connectivity between subsurface and surface temperatures in the equatorial Pacific^16^, missing remote impact of the Southern Ocean cooling that is also not reproduced in models^17,18^, among others. As multiple forcing mechanisms on the Pacific SST pattern change have been identified^12^, there may be other unidentified biases in the simulated forced response, which are not consistently reduced even in higher-resolution climate models^16^.

This study demonstrates, using analyses of observational data and large-ensemble historical simulations, that the past trends in the zonal SST gradient in the tropical Pacific can well be approximated by two patterns, one IPO and the other teleconnection mainly associated with the Atlantic multidecadal variability (AMV), the latter has a pattern bias in the model leading to a false tendency toward weakening of the SST gradient. This result aligns with previous studies that show a remote linkage between the AMV and the tropical Pacific SST^19,20^, but we further quantify that the bias in the AMV-related SST pattern change in the tropical Pacific reconciles at least partly the model-observation discrepancy in the zonal SST contrast trend. Importantly, the model reproduces the phase change of the AMV, which is suggested to have been controlled by external forcing such as aerosols^21–23^, indicating that improving remote pathways of the forced climate response is the key to better reproduce the tropical Pacific SST pattern change.

## Results

### Dominant patterns shaping the tropical Pacific SST trends

To identify the decadal-multidecadal variability in the tropical Pacific and the factors that shape multidecadal trends in the zonal SST gradient, ΔSST_*x*_ (see Methods for the definition), in observations, an empirical orthogonal function (EOF) analysis was performed on annual SST anomalies for 1920–2023, after removing a local long-term linear trend (Supplementary Fig. 1) and applying a 10-year low-pass filter (Methods). We primarily used the NOAA Extended Reconstructed SST version 5 (ERSSTv5) for our analyses, but similar results were obtained using two other observational SST datasets, HadISST1 and COBE-SST2 (Supplementary Fig. 2).

**Fig. 1:**
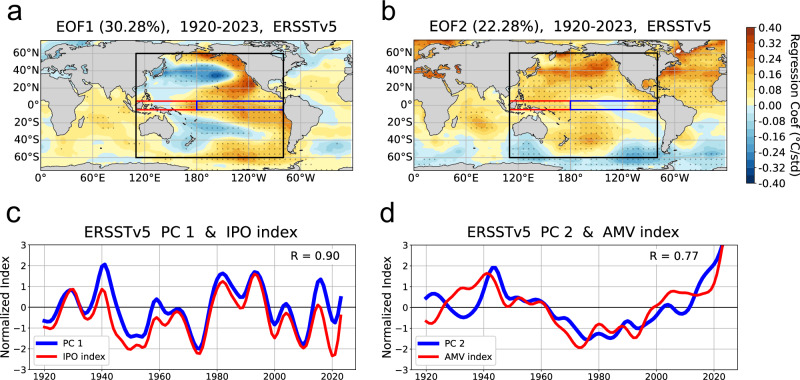
Leading patterns of the observed Pacific low-frequency SST variability from ERSSTv5 for 1920–2023. **a**, **b** Regression maps of 10-year low-pass filtered, detrended SST anomalies onto the first and second principal component (PC1 and PC2) time series, respectively. Regression coefficients are expressed in °C per standard deviation (°C/std) of the corresponding PC time series. The black box indicates the EOF analysis domain (60° S–60° N, 110° E–80° W), and the red and blue boxes indicate the regions used to define the zonal SST gradient. **c**, **d** PC1 and PC2 time series are shown by blue curves, respectively, with the low-pass filtered IPO and AMV indices shown by red curves. Correlation coefficients (R) between the PC time series and the corresponding index are shown in each panel. Stippling indicates regions statistically significant at the 95% level.

The first leading pattern (EOF1) accounts for approximately 30.3% of the total variance and exhibits a spatial pattern resembling the IPO (Fig. 1a, c). Indeed, the principal components (PCs) show decadal fluctuations, which is highly correlated with the tripole index (TPI), a conventional measure of the IPO^24^ (*r* = 0.90). Both the PC1 and TPI time series show a negative trend for the 44-year period between 1979 and 2022, contributing to strengthen the zonal SST gradient.

The second pattern (EOF2), explaining about 22.3% of the variance, displays a hemispherically asymmetric structure near the equator and fluctuates on multidecadal timescales (Fig. 1b, d). The EOF2 pattern accompanies warming in the extratropical Pacific, North Atlantic, and the Indian Ocean whereas cooling in the eastern equatorial Pacific and the Southern Ocean. It is not straightforward to understand the mechanism causing this pattern, but the PC2 time series shows a significant positive correlation with the AMV index defined by the basin-mean SST anomalies in the North Atlantic (*r* = 0.77), suggesting the AMV as being a major driver. Because of the prevailing multidecadal variability in the PC2 and the AMV index, the positive trend for 1979–2022 indicates the contribution of this pattern to strengthen the Pacific zonal SST gradient similar to EOF1.

**Fig. 2:**
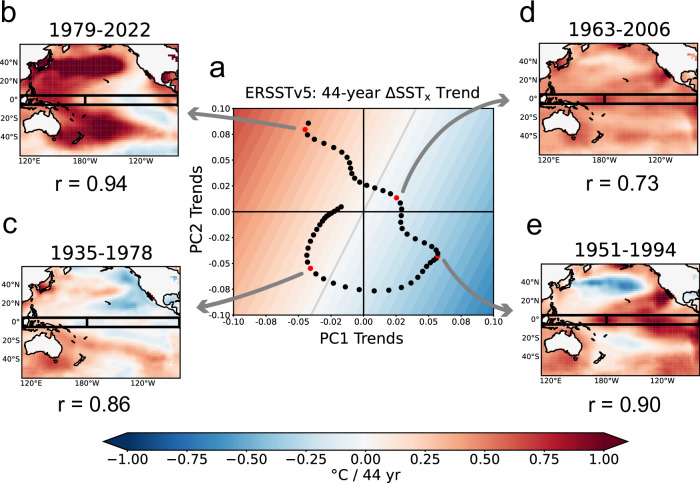
Observed 44-year trends in the equatorial Pacific zonal SST gradient (ΔSST_*x*_) varying with the phases of the IPO and AMV-related pattern. The dots in (**a**) show the trajectory of 44-year trends of the PC1 (*x*-axis) and PC2 (*y*-axis) from 1920 to 2023, imposed on the 44-year trends of the zonal SST gradient calculated by reconstructing the SST patterns using the two EOFs multiplied by the corresponding PC trends (shading). Four reconstructed SST trend maps for the 44-year periods indicated by red dots in (**a**) are shown in (**b**–**e**) for 1935–1978, 1951–1994, 1963–2006, and 1979–2022. Spatial correlation coefficients with the actual SST trend patterns over the same period are shown at the bottom.

Collectively, EOF1 (the IPO pattern) and EOF2 (the AMV-related pattern) account for over half of the Pacific SST variance, indicating that these two large-scale structures play key roles in shaping multidecadal trends over the tropical Pacific. Reconstructed SST anomalies by the two EOFs (Methods) illustrate how these two patterns jointly explain the ΔSST_*x*_ trend for a moving 44-year window. It is evident from the EOF patterns that the ΔSST_*x*_ trend is negative with a positive PC1 trend and positive with a positive PC2 trend (background shading in Fig. 2a). Depending on the phases of EOF1 and EOF2, the observed 44-year ΔSST_*x*_ trends fluctuate between strengthening and weakening during the period of 1920–2023 (dots in Fig. 2a). Indeed, the SST trend patterns reconstructed using the two EOFs for representative four periods on the trajectory (1935–1978, 1951–1994, 1963–2006, and 1979–2022) show a reversal of ΔSST_*x*_ between strengthening and weakening, and all these patterns are highly correlated with the actual trends (Fig. 2b–e and Supplementary Fig. 3).

In particular, the positive ΔSST_*x*_ trend, i.e., intensified climatological SST gradient, for the most recent period of 1979–2022 is found to occur due to a negative IPO trend and a large positive AMV trend, which has not happened for the entire period (Fig. 2a, b). The positive ΔSST_*x*_ trend for 1979–2022 shows 0.47 °C per 44 years, and 86.3 % of this trend is accounted for by the two EOFs (Supplementary Table 1). This implies that the multidecadal ΔSST_*x*_ trend primarily has only two degrees of freedom^9,25^, suggesting an importance to examine if climate models can reproduce these dominant patterns in order to understand the model-observation discrepancy.

### Possible source of the model-observation discrepancy

**Fig. 3:**
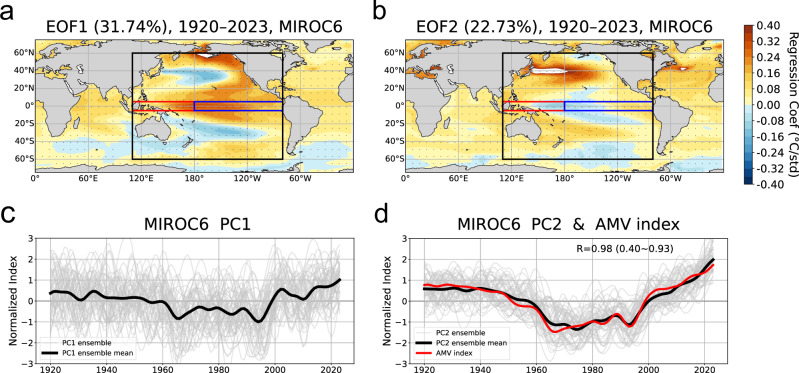
Leading patterns of the Pacific low-frequency SST variability for 1920–2023 in the MIROC6 large-ensemble simulations. The EOF patterns in (**a**) and (**b**) are the same as Fig. 1 but obtained from the whole ensemble containing 50 realisations. The PC time series in (**c**) and (**d**) are shown for individual members (thin grey curves) and their ensemble-mean time series are shown in thick black curves. The red curve in (**d**) indicates the ensemble-mean time series of the AMV index. R denotes the correlation between the ensemble-mean PC2 and the AMV index (R = 0.98), and the range of correlations computed separately for each ensemble member (0.40–0.93). Stippling indicates regions statistically significant at the 95% level.

To evaluate whether climate models can replicate the two leading patterns of SST variability identified in observations, we use a 50-member ensemble of historical simulations by MIROC6 as a primary example, supplemented by other CMIP models later (Methods). In MIROC6, the two leading EOFs, accounting for 31.7% and 22.7% respectively, show patterns that resemble observations (Fig.3), and yet noticeable differences exist in both spatial and temporal characteristics.

The EOF1 pattern exhibits an IPO-like structure and shows a high spatial correlation (*r* = 0.82) with observations (Fig.3a). Consistent with our understanding that the IPO is essentially an internal mode of variability, the PC1 time series shows a large ensemble spread, resulting in the ensemble-mean time series much smaller in magnitude than the individual members (Fig. 3c). This interpretation is further supported by the EOF analyses to the ensemble mean and the ensemble deviations, and the analysis of variance to the PC time series, which shows that 78.4% of the EOF1 is internally generated (Supplementary Fig. 4 and Supplementary Table 2; see Methods).

However, there exists a positive trend in the ensemble mean during recent decades, which may have contributed to weaken ΔSST_*x*_ in MIROC6. Indeed, the amplitudes of the IPO as measured by the standard deviation of PC1 time series are comparable between the observations and MIROC6, but the PC1 trend for 1979–2022 in MIROC6 is clearly shifted toward a positive value and does not capture the observed negative trend (Supplementary Fig. 11a, c, e). Given that the ensemble-mean trend represents a forced response, the above discrepancy suggests a potential bias in the forced mechanism that projects on a positive phase of the IPO and erroneously weaken ΔSST_*x*_ in MIROC6.

The EOF2 in MIROC6 exhibits a subtle but important difference from the observed counterpart in the spatial structure although their global patterns are positively correlated (*r* = 0.55). Namely, the equatorial cooling signal is shifted westward compared to the observations and warming appears in the eastern Pacific instead (Fig.3b). Due to this zonal distortion of the SST anomalies, the phase change in EOF2 would not much contribute to the ΔSST_*x*_ trends unlike observations. Nevertheless, the corresponding PC2 time series shows a remarkable similarity to the observed PC2 in terms of the phase change: positive before the 1960s and after the 2000s and negative in between (Fig. 3d). We regard the simulated EOF2 pattern as representing the global teleconnection associated with the AMV as in the observed EOF2 because of a very high correlation between the ensemble means of PC2 and the AMV index (*r* = 0.98). The AMV-related variability is partly coherent with multidecadal changes in the global-mean SST, which may be an important part of the model-observation discrepancy (see Methods). The coherence of PC2 across members results in a large ensemble-mean signal and an enhanced PC2–AMV correlation (Fig. 3d), accompanied by forced warming over the North Atlantic in the ensemble-mean EOF and with 67.6% of the EOF2 being forced (Supplementary Fig. 4 and Supplementary Table 2), consistent with previous studies^21–23^.

Because of the discrepancy in the EOF2 pattern in the tropical Pacific, the ΔSST_*x*_ trend is less dependent on the phase of the EOF2 in MIROC6 unlike observations (shading in Supplementary Fig. 5). As the EOF reconstruction similarly applies to the model results (Supplementary Fig. 6), this result indicates that the AMV-related teleconnection processes that cause the SST pattern to change in the tropical Pacific have a bias in MIROC6, which could be a source of the model-observation discrepancy in the ΔSST_*x*_ trend. This bias appears to be temporally robust in MIROC6, as the AMV-related teleconnection pattern remains broadly stable across different 44-year windows (see Methods).

Previous studies have shown coupled dynamical pathways by which the AMV can influence the tropical Pacific SST pattern. One is the extratropical teleconnection originated from the North Atlantic warming that induces anomalous high pressure over the North Pacific, which then modulates the PDO^26,27^. This may further extend to the western subtropical Pacific that acts to strengthen the zonal SST contrast and the trade wind near the equator^19^. Another mechanism is a tropical teleconnection triggered by the subtropical North Atlantic warming from which the coupled atmosphere-ocean process propagates westward and eastward, reaching the equatorial Pacific and also strengthen the zonal SST gradient^27,28^. Our analysis, however, suggests that the teleconnection mechanism is better explained by a hemispheric energy constraint coupled with the tropical atmosphere-ocean feedback.

Atmospheric anomalies regressed onto the observed PC2 time series reveal an enhanced surface wind speed over the central-eastern equatorial Pacific (Supplementary Fig. 7a), which would cool the ocean surface by the wind–evaporation–SST (WES) feedback^29,30^. The accelerated equatorial wind may partly arise from the intensified zonal SST gradient (Fig.1b) but happens primarily due to the anomalous cross-equatorial winds associated with a northward shift of the Inte-Tropical Convergence Zone (ITCZ) (Supplementary Fig.7c, e). This ITCZ shift can be explained as a Hadley-cell adjustment to a hemispheric thermal contrast, i.e., warming in the North Atlantic, as demonstrated by many studies^31–33^. In contrast, MIROC6 shows enhanced wind speed over the western equatorial Pacific and the trade wind is weakened over the central-eastern Pacific (Supplementary Fig. 7b). The model response lacks the cross-equatorial flow because the ITCZ does not shift (Supplementary Fig. 7d, f). As a result, the model fails to reproduce the feedbacks necessary to reinforce the Pacific zonal SST contrast.

### Reconciling the model-observation discrepancy

A central question arising from the preceding analyses is to what extent the biased Atlantic–Pacific teleconnection and a forced variability within the tropical Pacific explain the failure of climate models to reproduce the recent zonal SST gradient strengthening. To quantify these effects, we conduct a simple reconstruction test to correct the biases (see Methods). Specifically, we (i) replace the EOF2 spatial pattern in MIROC6 with the observed pattern while retaining the model’s PC2 time series (correction to the AMV teleconnection pattern bias), (ii) remove the ensemble-mean component of the PC1 time series by assuming that the forced component in EOF1 has a bias (forced-IPO removal), and (iii) evaluate their combination. The true forced signal in the IPO is unknown, so the test (ii) gives a null hypothesis. We then compare the reconstructed ΔSST_*x*_ trends for 1979–2022 with the observed trends (cf. Fig. 2b).

**Fig. 4:**
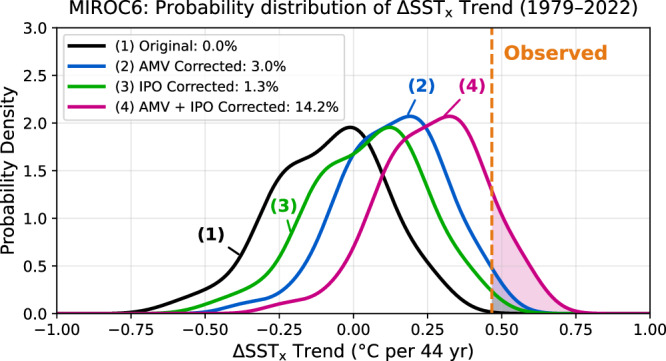
Probability distribution of the ΔSST_*x*_ trends for 1979–2022 in the MIROC6 large ensemble. Gaussian kernel-density estimates are shown for four MIROC6 large-ensemble data sets: (1) the original distribution drawn from the raw lowpass-filtered SST fields (black), (2) the AMV-corrected distribution, in which the model EOF2 pattern is replaced by the observed EOF2 pattern (blue), (3) the IPO-corrected distribution, in which the PC1 ensemble mean is removed (green), and (4) the AMV + IPO-corrected distribution, in which both corrections are applied (magenta). The dashed orange line (0.47 °C per 44 yr) marks the observed trend, and the shading indicates the fractional area exceeding the observation (0.0, 3.0, 1.3, and 14.2%, respectively).

Applying this combined correction method to the MIROC6 large ensemble results in a clear displacement in the probability distribution function (PDF) of the ΔSST_*x*_ trends, which agree better with observations (Fig. 4). The original PDF without the correction shows that virtually none of the ensemble members can reproduce the observed gradient strengthening (black curve). When we correct the pattern bias in the AMV teleconnection, the probability increases to 3.0% (blue curve). The removal of the forced IPO signal has a smaller effect and acts to increase the probability to 1.3% (green curve). After applying both corrections, the ensemble-mean ΔSST_*x*_ trend changes sign from negative (weakening) to positive (strengthening), and the probability that exceeds the observed strengthening increases to 14.2% (red curve and shading in Fig. 4). This hypothetical result suggests that if the model can eliminate the two kinds of biases, i.e., AMV teleconnection and the forced IPO, then the observed strengthening of the zonal SST gradient for 1979–2022 would have occurred more frequently in the ensemble historical simulations.

### Multi-model analysis

We repeated the EOF analysis using large-ensemble historical runs by nine other CMIP models for 1920–2023 (Methods), and the resultant EOF patterns and PC time series are shown in Supplementary Figs. 8 and 9. As in MIROC6, in all nine CMIP models the two leading EOFs closely resemble the observed IPO and AMV-related patterns but the order appears swapped in some models. We successfully classified them into the IPO and AMV-related variability using their spatial and temporal correlations with the observed EOF1 and PC2 (Methods and Supplementary Fig. 10). In MIROC6, MPI-ESM-LR and MPI-ESM1-2-LR, the EOF1 and EOF2 represent IPO and the AMV-related pattern, respectively, as in observations, whereas in the other seven models the order is swapped and yet each of the two EOFs is corresponding to the observed EOF. While the IPO pattern is well represented in models, the AMV-related teleconnection pattern in the tropical Pacific is commonly biased despite the time evolution being well reproduced (Supplementary Figs. 8, 9, 10).

A comparison of the PC standard deviations between observations and models shows that the IPO amplitude is realistic but the variance of the AMV-related variability is overestimated in some models (Supplementary Fig. 11a, b), which explains why the order of the two EOFs is swapped. In contrast, the PC trends for 1979–2022 show a large inter-model difference for the IPO, which does not cover the observed negative trend well, and a good agreement for the positive AMV trend between observations and models (Supplementary Fig. 11c, d). Nevertheless, the ∆SST_*x*_ trends for the same period reconstructed from each of the EOFs reveal a systematic underestimate of the observed trend related with the AMV (Supplementary Fig. 11e, f), suggesting a common multi-model bias in the teleconnection pattern as identified in MIROC6.

**Fig. 5:**
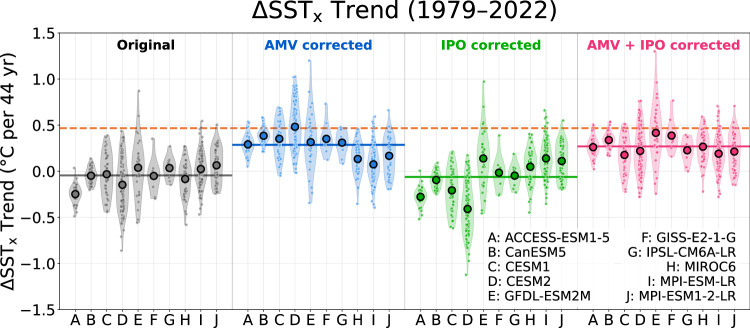
The ΔSST_*x*_ trends for 1979–2022 in the 10 CMIP large ensembles. For each model (A–J; listed in the panel), black, blue, green and magenta violins show the ensemble distribution of ΔSST_*x*_ from the Original, AMV-corrected, IPO-corrected and AMV + IPO-corrected SST fields, respectively. Violin widths indicate Gaussian kernel-density estimates of the ensemble distributions. Ensemble mean and individual members for each model are shown by large and small dots, respectively. The orange dashed horizontal line denotes the observed ΔSST_*x*_ trend. Coloured horizontal segments behind each block of violins indicate the MMM trend (averaged over members within each model, then equally over models). Details of the EOF-based corrections and model grouping are given in Methods.

**Fig. 6:**
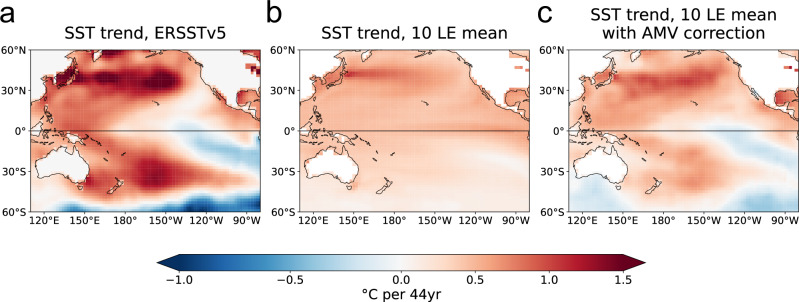
Spatial patterns of SST trends for 1979–2022 in observations and MMMs. The observed SST trend (**a**) replicated from Fig. 2b and the MMM SST trends from the 10 CMIP large ensembles (**b**–**c**), in which the AMV-related teleconnection is corrected in (**c**) as Fig. 5.

Multi-model results of the reconstruction test for the 1979–2022 ∆SST_*x*_ trends are summarised in Fig. 5 (individual PDFs are shown in Supplementary Fig. 12), which supports that the arguments based on MIROC6 apply to other models. Namely, the SST gradient tends to be intensified or less weakened after the correction of the AMV-related teleconnection pattern (blue bars) compared to the original results (black bars) in all models. Indeed, the multi-model mean (MMM) ∆SST_*x*_ trend was nearly zero (−0.05 °C per 44 years), which increases to 0.29 °C per 44 years, closer to the observed trend of 0.47 °C per 44 years. A possible forced trend in IPO is model-dependent, so that the removal of the ensemble-mean IPO component does not show a systematic improvement (green bars). It is therefore not surprising that the combined corrections mainly reflect the effect of correcting the AMV-related pattern in the tropical Pacific (magenta bars). A comparison of the SST trend patterns for 1979–2022 confirms that the MMM after correcting the AMV-related teleconnection can reproduce the observed change well (Fig. 6). Overall, these results support our conclusions that biases in remote teleconnections arising from the AMV are an important source of model discrepancy in the ∆SST_*x*_ trends.

## Discussion

The pattern bias in the EOF2 that represents the teleconnection from the AMV to the Pacific appears to arise from the absence of the ITCZ shift in the Pacific in response to the North Atlantic warming. In observations, cross-equatorial wind anomalies associated with the northward shift of the ITCZ accompany the intensified equatorial trade winds that can cool the central-eastern Pacific via a wind-induced evaporation (Supplementary Fig. 7). In MIROC6, however, the corresponding ITCZ shift and wind response are not reproduced well. Possible reasons for this failure are the tropical Pacific mean-state biases. In particular, a too-weak cold tongue and biased ITCZ may distort the atmospheric response in the tropical Pacific^14,15,34,35^. Such a distorted response could weaken or shift the Walker circulation and cross-equatorial wind anomalies triggered by North Atlantic warming^19,20,27,28^. Although our analysis does not isolate the full tropical pathway, previous studies have demonstrated the importance of tropical basin coupling^36^ which is likely underestimated in climate models and may partly explain the model-observation discrepancy in the EOF2 pattern.

Observed PC1 exhibits a negative trend after the late 1970s, yet most members in the MIROC6 large ensemble show a positive PC1 trend over the same period (Supplementary Fig. 11c). The forced component embedded in the EOF1 can be extracted by the regression of ensemble-mean SST onto the ensemble-mean PC1 in MIROC6 (Supplementary Fig. 4a). The pattern shows a slight reduction of ΔSST_*x*_ and a broad warming in the northern extratropics, the latter feature being different from the internally generated component (Supplementary Fig. 4b). Given multiple forcing mechanisms are at work for the recent ΔSST_*x*_ trend^12^, it is difficult to attribute this forced pattern to a single external driver. Previous studies have suggested that the IPO or PDO was partly forced by aerosols^37–39^ and an ocean thermostat mechanism linked to rising greenhouse forcing^25^. However, the sign of the ensemble-mean IPO trends for 1979–2022 varies across 10 models (Supplementary Fig. 11c), which indicates the forced component in the IPO may not be represented robustly in current climate models.

One important candidate is the recent Southern Ocean (SO) cooling, which has contributed to cool the eastern tropical Pacific and thereby to intensify ΔSST_*x*_^17,18,40^. In our observational analysis, the SO SST index, defined as the SST anomaly averaged in the Pacific side of 75°–45° S and 110°–280° E, shows decadal variability and is positively correlated with the PC1 time series (*r* = 0.64; Supplementary Fig. 13a). A similar link between the PC1 and the SO SST variability is found in the MIROC6 large ensemble, where the ensemble-mean correlation is *r* = 0.87 (range 0.29–0.83; Supplementary Fig. 13b), but the ensemble fails to reproduce the recent SO cooling trend (Supplementary Fig. 13c). The lack of the recent SO cooling in the historical simulation is a common error in climate models^9,10^, and it may partly explain the forced positive IPO trend in models, consistent with our assumption that the simulated forced IPO signal is biased.

A critical assumption in this study is that the two leading SST EOFs identified in observations are similarly obtained from individual climate model simulations. This condition is satisfied in 10 large ensembles including MIROC6, but may not necessarily be the case for other models. In some simulations, the ensemble size is relatively small, which makes it harder to cleanly separate the IPO and AMV-related signals and to identify the two modes in the same way as in observations. Moreover, our diagnostics alone cannot determine whether the Atlantic–Pacific teleconnection bias mainly arises from errors in the tropical Pacific mean state or from other dynamical processes. Pacemaker-type experiments that prescribe observed North Atlantic SST trends may not resolve this issue given that the teleconnection pattern away from the Atlantic is biased due perhaps to errors in the processes and mean states in the tropical Pacific. Future work using targeted experiments that combine Atlantic SST forcing with corrections to the tropical Pacific mean state (e.g., using flux adjustments) would be promising to verify the mechanism proposed in this study^35^. Our approach should also be revisited when new large ensembles become available in the CMIP Phase 7.

## Methods

### Tropical Pacific zonal SST gradient (ΔSST_*x*_)

Following ref. ^41^, ΔSST_*x*_ is defined as the difference in area-mean annual SST anomalies between the western equatorial Pacific (5°S–5°N, 110°E–180°) and the eastern equatorial Pacific (5°S–5°N, 180°–80°W).

### Definition of low-frequency anomalies

All monthly data are converted to annual means by weighting each month according to its number of days. The 1981–2010 climatology is then subtracted from each annual mean to produce anomalies, and a 10-year low-pass Butterworth filter (2nd order) is applied to remove high-frequency variability.

### Detrending and EOF analysis

Prior to the EOF calculations, a local linear trend for the entire record of 1920–2023 was removed at each grid to exclude the long-term global warming, leaving emphasis on decadal and multidecadal variability. The detrended anomalies in the Pacific domain (60°S–60°N, 110°E–80°W) were used for the EOF analysis. In the EOF analysis of CMIP models, ensemble members from 1920 to 2023 in each model were concatenated into a single dataset before performing the EOF analysis. This approach produces two leading EOF patterns common to the ensemble members and associated PC time series for individual members. For COBE-SST2, we applied a variance-matching procedure by rescaling the local standard deviation at each grid according to the ratio between ERSSTv5 and COBE-SST2 because the leading two EOFs were mixed and not well separated in the original data.

### Sensitivity to detrending methodology

To assess the extent to which the EOFs are sensitive to the detrending method, we repeated the analysis using an alternative approach with which SST anomalies at each grid regressed onto the global-mean (60°S–60°N) SST time series have been removed^42,43^ (Supplementary Fig. 14). The observed ΔSST_*x*_ trend for 1979–2022 is similar between the two detrending methods (0.43 and 0.42 °C/44 yr, respectively), and the EOF2 patterns and PC2 time series also do not reveal much difference (spatial correlation of *r* = 0.76). In MIROC6, however, the multidecadal ensemble-mean PC2 variability is strongly damped with an alternative detrending (Supplementary Fig. 14f) and the correlation between the ensemble-mean PC2 and the AMV index is also reduced from 0.98 to 0.63 with a large ensemble spread (Supplementary Fig. 15). Therefore, we use the linear detrending in the main analysis to extract the AMV-related variability.

### Attribution of modes of variability in the large ensemble

We separated the large-ensemble SST anomalies into the ensemble mean (forced response) and deviations from the ensemble mean (internal variability) and then repeated the EOF analysis (Supplementary Fig. 4). The EOF1 to the forced component shows warming in the North Pacific and Atlantic and a weakened zonal SST contrast in the equatorial Pacific. This pattern resembles the AMV-related pattern shown in Fig. 3b, and the PC time series are similarly indicating the multidecadal signal. In contrast, the EOF1 to the internal component clearly exhibits the IPO pattern consistent with Fig. 3a. This result supports our arguments that the IPO (Fig. 3a) is mainly internally generated whereas the AMV-related variability (Fig. 3b) is largely induced by external forcing. To further quantify the forced versus internally generated components in the MIROC6 large ensemble, the “analysis of variance” (ANOVA)^44^ was applied to the EOF results shown in Fig. 3. In ANOVA, the PC time series is decomposed into the ensemble mean and deviations from the ensemble mean. The variances using the respective components, denoted as ${\sigma }_{{\rm{F}}{\rm{orced}}}^{2}$ and ${\sigma }_{{\rm{Int}}}^{2}$, are then divided by the variance of the raw PC time series, ${\sigma }_{{\rm{Total}}}^{2}$, leading to the fractional contribution of the forced response and internal variability. The results presented in Supplementary Table 2 consistently show that the EOF2 (AMV-related variability) is predominantly forced (67.6%) whereas the EOF1 (IPO) is mostly induced by internal variability (78.4%).

### Regression and PDF

All regression analyses were performed by regressing low-pass filtered variables (e.g., SST, precipitation) onto the PC time series obtained from the EOF analysis. These variables were not detrended prior to the regression, as it had negligible impact on the results. The statistical significance of the regression coefficients was assessed using a two-sided Student’s t-test, with autocorrelation accounted for using the effective degrees of freedom^45,46^. The PDF is estimated using a Gaussian kernel density estimation, with the bandwidth selected according to Scott’s rule. The proportion of ensemble members exceeding the observed trend is calculated as the area under the density curve beyond the observed value.

### Stationarity of the AMV-related teleconnection

To assess the stationarity of the AMV-related teleconnection, we repeated the SST regression onto PC2 using 44-year windows within 1920–2023 and computed the spatial correlation between each window-specific regression map and the corresponding full-period pattern over the Pacific domain (60°S–60°N, 110°E–280°E) (Supplementary Fig. 16). For MIROC6, concatenated SST anomalies and PC2 time series from all ensemble members were used in each window. The resulting rolling correlations indicate that the pattern is broadly stable in MIROC6, with values mostly near 0.9, whereas in observations the similarity to the full-period pattern increases toward recent decades.

### Observational data

We used observed monthly SSTs derived from three gridded products: the NOAA Extended Reconstructed SST version 5 (ERSSTv5)^47^, the Centennial in situ Observation-Based Estimates version 2 (COBE-SST2)^48^, and the Hadley Centre Sea Ice and SST dataset version 1.1 (HadISST1)^49^. The ERSSTv5 data cover the period 1854–2023 with a 2° × 2° resolution, COBE-SST2 spans 1850–2023 at 1° × 1°, and HadISST1 provides data from 1870–2023 at 1° × 1° resolution. We re-gridded COBE-SST2 and HadISST1 data to the 2° × 2° resolution to match ERSSTv5. Surface wind data, including 10-m wind vectors and wind speed, were derived from the ERA5 reanalysis^50^ for 1940–2023. Sea-level pressure fields are also taken from ERA5. Monthly precipitation data were obtained from the Global Precipitation Climatology Project version 2.3 (GPCP)^51^ for 1979–2024. The SST datasets were used for the main analyses, while the wind, sea-level pressure, and precipitation datasets were used only in the Supplementary Information.

### Interdecadal Pacific Oscillation (IPO) index

The IPO is represented by the Tripole Index (TPI) following Ref. ^24^, defined as TPI = SSTA(R2) – 0.5 × [SSTA(R1) + SSTA(R3)], where SSTA denotes annual SST anomalies averaged over the following regions: R1 (25°N–45°N, 140°E–145°W), R2 (10°S–10°N, 170°E–90°W), and R3 (50°S–15°S, 150°E–160°W). The resulting TPI time series is detrended over 1920–2023 and normalised by its standard deviation.

### Atlantic Multidecadal Variability (AMV) index

The AMV index is defined as the area-weighted mean annual SST anomaly over the North Atlantic (0–70°N, 80°W–0°), consistent with NOAA’s AMO definition (https://psl.noaa.gov/data/timeseries/AMO/). The time series is linearly detrended over 1920–2023 and normalised by its standard deviation. For the detrending sensitivity test, the AMV index is detrended consistently with the SST fields, using either linear detrending or a detrending method based on global-mean SST.

### Large ensemble climate simulations

Large-ensemble (LE) climate simulations obtained from 10 CMIP5 and CMIP6 models are analysed in this study. Each LE consists of many realisations made with the identical boundary conditions but integrated from slightly different initial conditions, thereby sampling a wide range of internal variability under the same external forcing. We primary used the MIROC6 LE (Model for Interdisciplinary Research on Climate version 6, CMIP6 generation) which consists of 50 members driven by the CMIP6 historical forcing up to 2014 and SSP2-4.5 thereafter. The model resolution is approximately 1.4° in the atmosphere and nominal 1° in the ocean. The ensemble data is available from JAMSTEC upon request^52,53^. The other nine LEs are: 40-member ACCESS-ESM1-5^54^, 50-member CanESM5^55^, 40-member CESM1^56^, 100-member CESM2^57^, 30-member GFDL-ESM2M^58^, 10-member GISS-E2-1-G LE^59^, 11-member IPSL-CM6A-LR^60^, 100-member MPI-ESM-LR LE^61^, 50-member MPI-ESM1-2-LR LE^62^. These simulations except CESM1, GFDL-ESM2M, MPI-ESM-LR follow the CMIP6 protocol and the data are publicly available (see Supplementary Text 1 for details).

### Reconstruction tests

An EOF-based reconstruction of tropical Pacific SST anomalies is used to examine how the potential errors in IPO and AMV-related variability influence the ΔSST_*x*_ trend for 1979–2022 (Figs. 4 and 5). For large-ensemble model simulations, annual SST anomalies for 1920–2023 are decomposed into a centennial-scale linear trend and a set of EOFs that are computed from the concatenated ensemble × time dimension. A baseline reconstructed SST field, ${{SST}}_{{\rm{r}}{\rm{econ}}}\left(x,t\right)$, is expressed as

1 $${{SST}}_{{\rm{r}}{\rm{econ}}}\left(x,t\right)={{SST}}_{{\rm{cent}}}\left(x,t\right)+\mathop{\sum }\limits_{k=1}^{N}{{PC}}_{k}(t) \,{{EOF}}_{k}(x)$$

where ${{SST}}_{{\rm{c}}{\rm{ent}}}\left(x,t\right)$ is the local linear trend for 1920–2023, ${{EOF}}_{k}(x)$ is the $k$-th EOF pattern, and $P{C}_{k}(t)$ is its associated time series. The number of EOFs is truncated at *N* = 104, which corresponds to the maximum degrees of freedom in observations, i.e., 104 years for 1920–2023 (note that we truncated at *N* = 2 for the background shading in Fig. 2). For each of the 10 LEs, the leading two EOFs are identified to represent IPO and the AMV-related variability like observations. Their patterns, denoted as ${EO}{F}_{{\rm{IPO}}}(x)$ and ${EO}{F}_{{\rm{AMV}}}\left(x\right)$, and associated PC time series, $P{C}_{{\rm{IPO}}}(t)$ and $P{C}_{{\rm{AMV}}}(t)$ are modified in the reconstruction test. A correction to the AMV-related pattern replaces ${EO}{F}_{{\rm{AMV}}}\left(x\right)$ in a model with the observed pattern ${{EOF}}_{{\rm{AMV}}}^{{\rm{obs}}}(x)$ while keeping $P{C}_{{\rm{AMV}}}(t)$ unchanged:

2 $${{SST}}_{\mathrm{mod},{\rm{AMV}}}(x,t)=	 {{SST}}_{{\rm{r}}{\rm{econ}}}(x,t)-P{C}_{{\rm{AMV}}}(t)\,{{EOF}}_{{\rm{AMV}}}(x) \\ 	+P{C}_{{\rm{AMV}}}(t)\,{{EOF}}_{{\rm{AMV}}}^{{\rm{obs}}}(x)$$

On the other hand, the IPO correction removes the ensemble-mean (forced) component in $P{C}_{{\rm{IPO}}}(t)$, denoted as $\left\langle P{C}_{{\rm{IPO}}}(t)\right\rangle$ and therefore the corrected reconstruction will be

3 $${{SST}}_{\mathrm{mod},{\rm{IPO}}}\left(x,t\right)=	 {{SST}}_{{\rm{orig}}}\left(x,t\right)-P{C}_{{\rm{IPO}}}\left(t\right)\,{{EOF}}_{{\rm{IPO}}}\left(x\right) \\ 	+P{C}_{{\rm{IPO}}}^{\mathrm{int}}\left(t\right)\,{{EOF}}_{{\rm{IPO}}}\left(x\right)\,$$

where

4 $$P{C}_{{\rm{IPO}}}^{\mathrm{int}}(t)=P{C}_{{\rm{IPO}}}(t)-\left\langle P{C}_{{\rm{IPO}}}\left(t\right)\right\rangle \,$$

represents internally generated component of IPO. The combined reconstruction can be made by using both corrections. These EOF-based reconstructions provide a “what-if” test for the ΔSST_*x*_ trend in climate models when biases in the IPO and AMV-related variability in the models are eliminated and can be done without performing additional simulations.

### Classification of IPO and the AMV-related pattern

To clarify the correspondence between the observed and simulated modes of variability, we use two metrics: observed EOF1 as the IPO pattern and observed PC2 as the AMV index. We made this choice because the PC1 time series, largely due to internal variability, should not reveal a significant correlation between observations and models whereas the simulated EOF2 patterns may be biased. Therefore, spatial correlations over the Pacific domain (60°S–60°N, 110°E–80°W) are calculated between the observed EOF1 and the two EOFs for each model, and temporal correlations are calculated between the two PC time series and the observed PC2 (Supplementary Fig. 10). When the spatial correlation with the observed EOF1 exceeds 0.5, we classify the simulated EOF as IPO. Likewise, when the temporal correlation with the observed PC2 time series exceeds 0.5, the corresponding EOF is regarded as an AMV-related variability. Models with the two EOFs appearing in the same order as observations are MIROC6, MPI-ESM-LR, and MPI-ESM1-2-LR, and others (ACCESS-ESM1-5, CanESM5, CESM1, CESM2, GFDL-ESM2M, GISS-E2-1-G, and IPSL-CM6A-LR) reveal their EOF1 representing the AMV-related variability as its variance is overestimated (Supplementary Fig. 11b).

### Southern Ocean (SO) SST index

SO SST index is computed as the area-mean annual SST anomaly over the Pacific sector of the Southern Ocean (45°S–75°S, 110°E–280°E). The time series is detrended over 1920–2023 and normalised by its standard deviation prior to analysis.

## Supplementary information

Supplementary Information

Transparent Peer Review file

## Data Availability

All observational and reanalysis datasets used in this study are publicly available from their respective repositories. ERSSTv5 (NOAA NCEI; https://www.ncei.noaa.gov/products/extended-reconstructed-sst) and HadISST1 (Met Office Hadley Centre; https://www.metoffice.gov.uk/hadobs/hadisst/) were used for sea-surface-temperature analyses. ERA5 reanalysis (Copernicus Climate Data Store; https://cds.climate.copernicus.eu/) and GPCP precipitation (NOAA PSL; https://psl.noaa.gov/data/gridded/data.gpcp.html) were used for atmospheric fields. CMIP large-ensemble simulations are available via the ESGF data portal (https://esgf-node.llnl.gov/), including ACCESS-ESM1-5, CanESM5, CESM1, CESM2, GFDL-ESM2M, GISS-E2-1-G, IPSL-CM6A-LR, MIROC6, MPI-ESM-LR and MPI-ESM1-2-LR. All dataset identifiers, experiment labels and version numbers are listed in the References and in the accompanying data catalogue.

## Code availability

The code and the processed data used for the analyses and figure generation in this study are available at Zenodo (10.5281/zenodo.18670279)^63^.
