# Supplementary material for: North Atlantic influence reconciling model-observation discrepancy in the tropical Pacific warming pattern
Source: Nat Commun. 2026 May 28;17:6930. doi: 10.1038/s41467-026-73763-0 (PMC13389187; doi:10.1038/s41467-026-73763-0)
Supplement: Supplementary file 1 — Supplementary Information [file 41467_2026_73763_MOESM1_ESM.pdf]

Supplementary Information

North Atlantic influence explaining model-observation discrepancy in the tropical Pacific warming pattern

Yueh-Chi Lin[1*], Masahiro Watanabe[1*]

1: Atmosphere and Ocean Research Institute, University of Tokyo, Chiba, Japan

*Corresponding authors: Yueh-Chi Lin, yueh-chi@aori.u-tokyo.ac.jp; Masahiro Watanabe, hiro@aori.u-tokyo.ac.jp

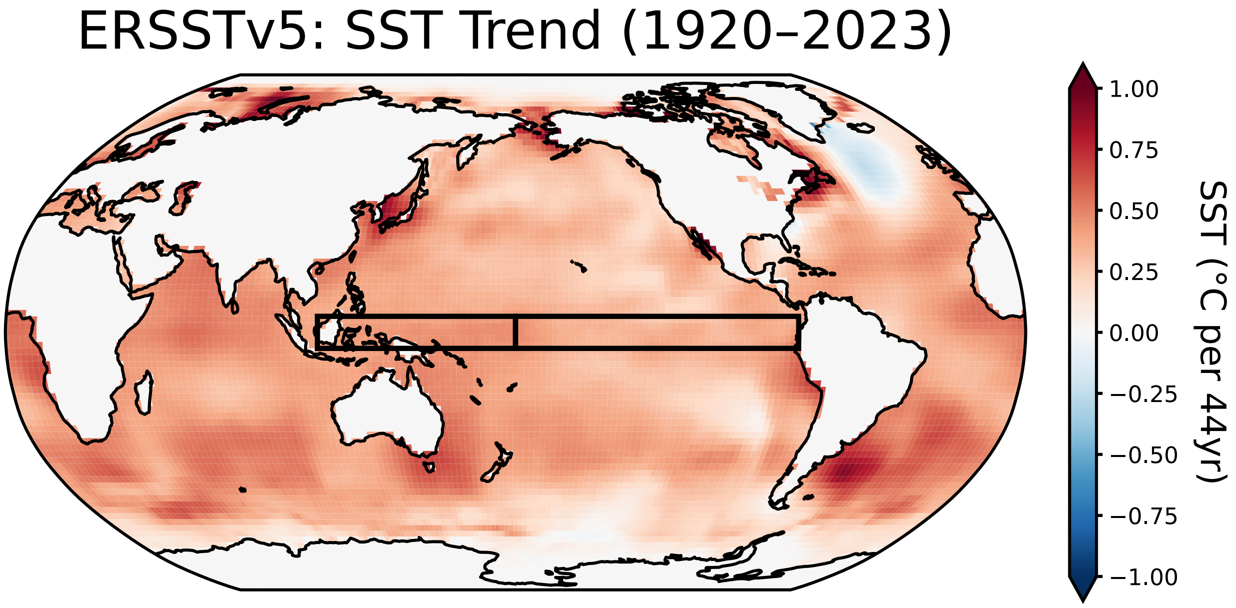

Supplementary Figure 1 | Observed pattern of SST trends for 1920–2023.

The linear trends (°C per 44 years) are calculated using annual-mean SST anomalies from ERSSTv5. The black boxes indicate the Western Pacific (WP: 5°S–5°N, 110°E–180°E) and Eastern Pacific (EP: 5°S–5°N, 180°–80°W) regions used to calculate the zonal SST gradient (WP minus EP).

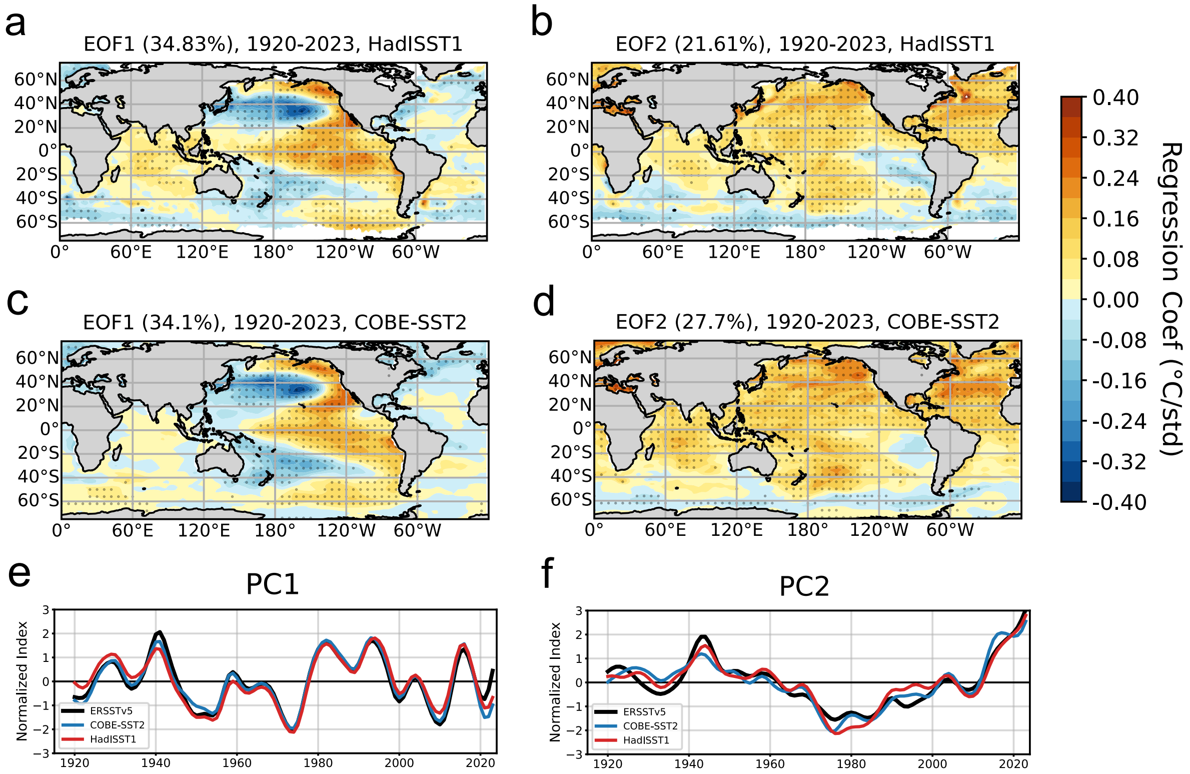

Supplementary Figure 2 | Leading patterns of the observed Pacific low-frequency SST variability for 1920–2023 in HadISST1 and COBE-SST2.

Same as Fig. 1 but for (a, b) HadISST1 and (c, d) COBE-SST2. Local standard deviations in COBE-SST2 have been adjusted to match ERSSTv5 (see Methods). Panels show regression maps of SST anomalies onto PC1 (a, c) and PC2 (b, d), with stippling indicating regions statistically significant at the 95% level. (e, f) PC1 and PC2 time series from ERSSTv5 (black), COBE-SST2 (blue), and HadISST1 (red), respectively.

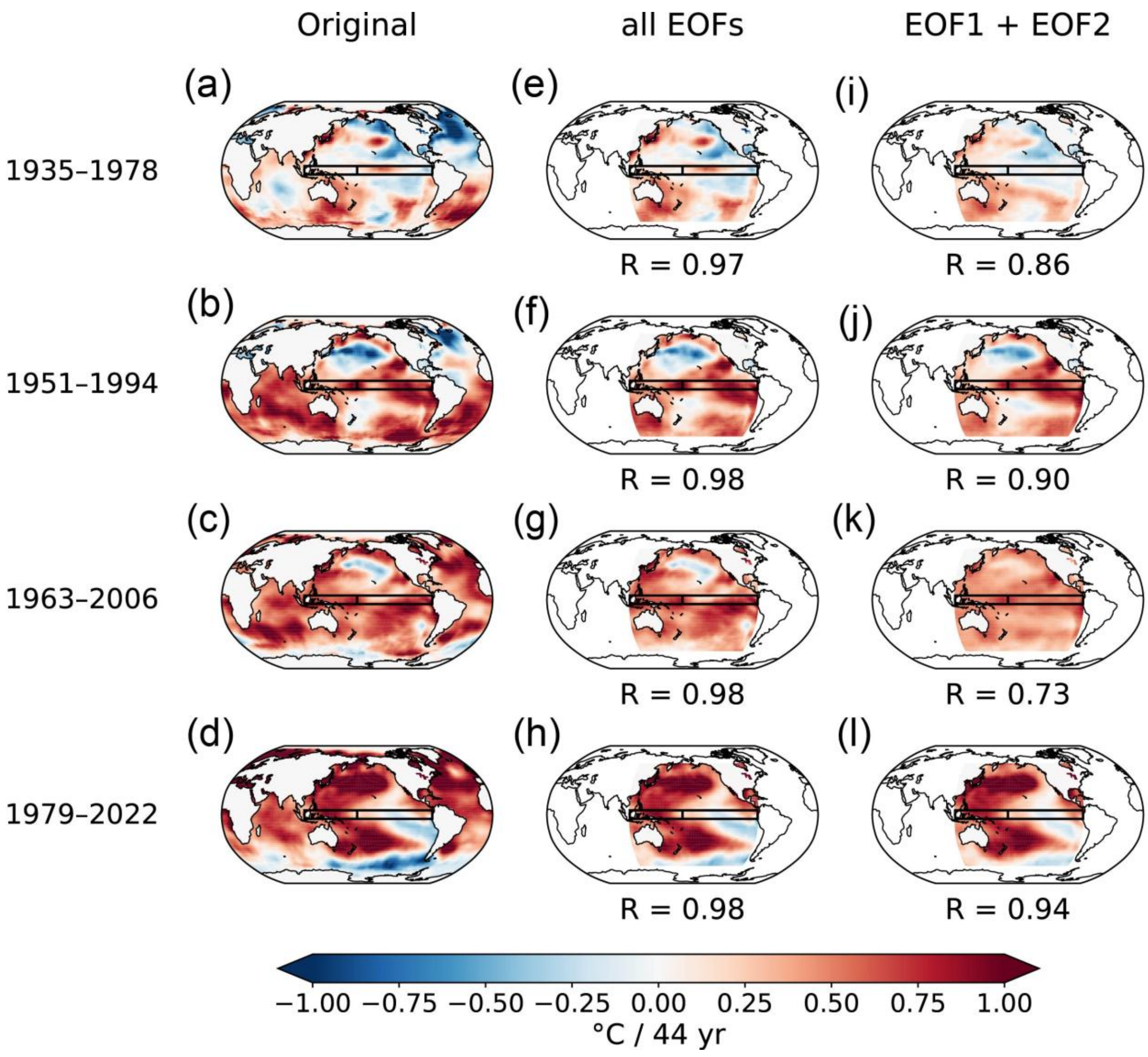

Supplementary Figure 3 | Observed and EOF-reconstructed 44-year SST trends in ERSSTv5. (a–d) Linear trends (°C per 44 yr) of annual-mean SST anomalies for 1935–1978, 1951–1994, 1963–2006 and 1979–2022; black rectangles indicate the western (WP) and eastern (EP) equatorial Pacific boxes. (e–h) Trends reconstructed from the sum of all EOFs plus the long-term trend (1920–2023). (i–l) Trends reconstructed with only EOF1 and EOF2 added to the long-term trend. Spatial correlations between the observed and reconstructed trends are shown at the bottom of panels (e-l).

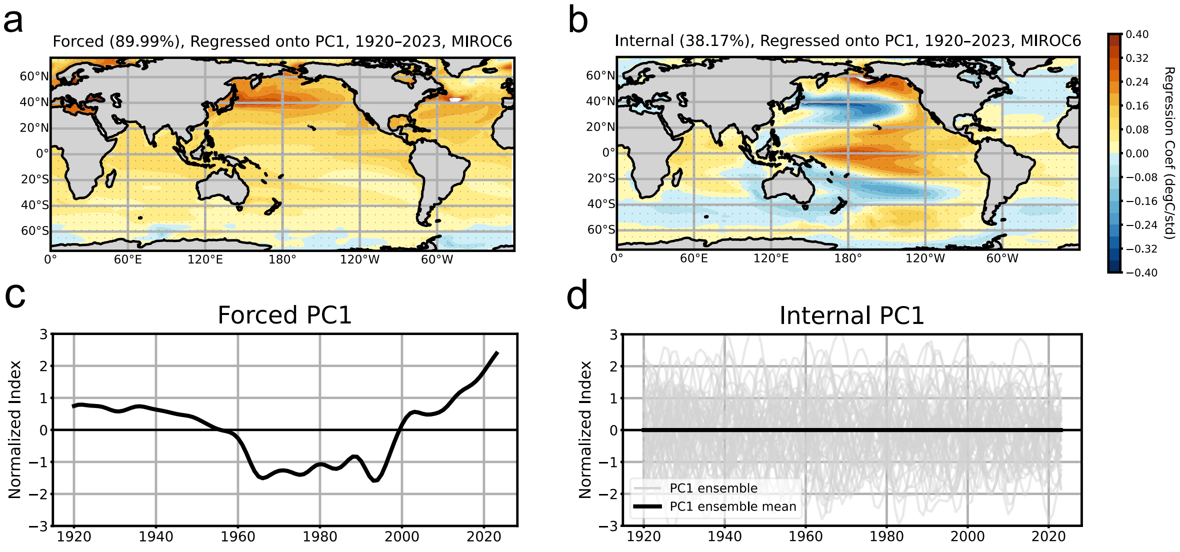

Supplementary Figure 4 | PC1 from EOFs of ensemble-mean and ensemble-deviation SST anomalies in the MIROC6 large ensemble.

(a) Regression of ensemble-mean SST anomalies for 1920–2023 onto the PC1 derived from the ensemble-mean field and (b) as in (a) but using SST deviations and the PC1 derived from the deviation field. (c, d) Corresponding PC1 (black: ensemble mean; grey: individual members). Stippling indicates significance at the 95% level. See Methods (*"Attribution of modes of variability in the large ensemble"*).

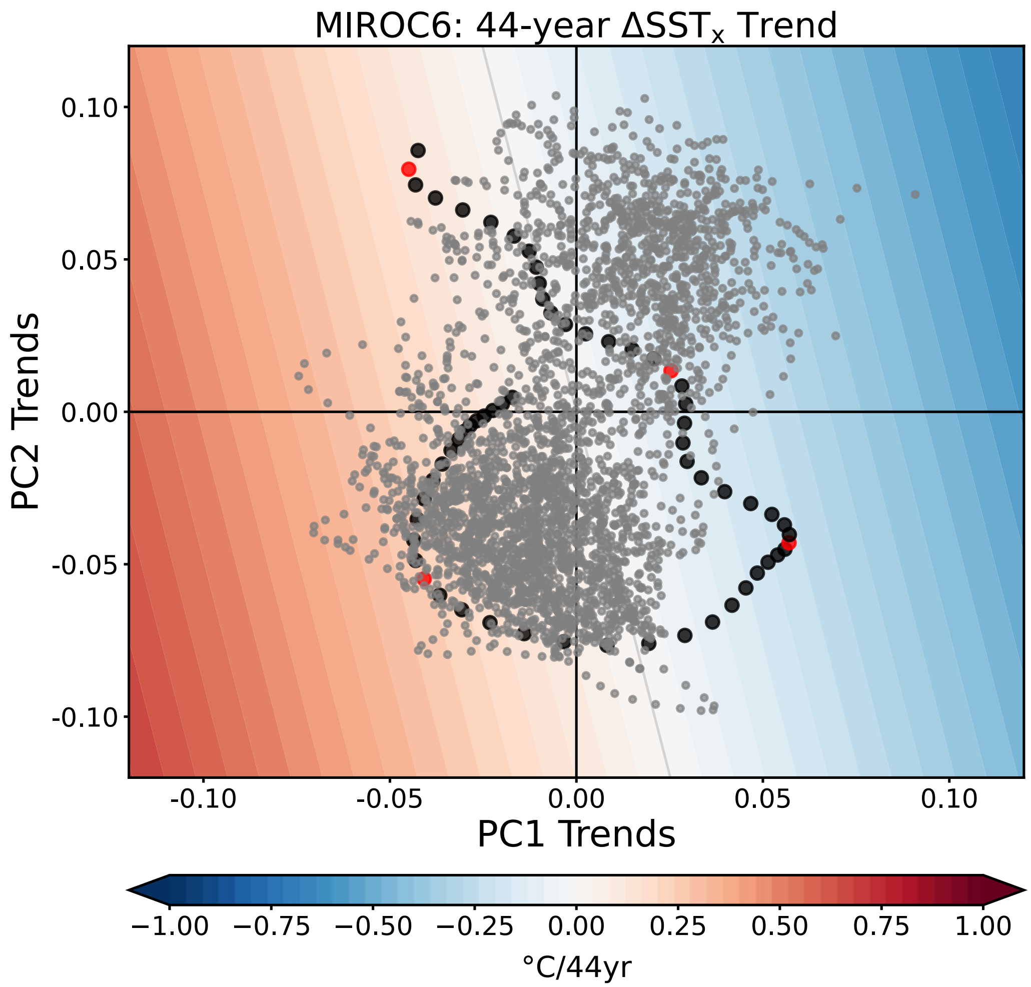

Supplementary Figure 5 | 44-year trends in ΔSST$_x$ varying with the phases of the IPO and AMV-related patterns in the MIROC6 large ensemble.

The background shading shows ΔSST$_x$ (°C per 44 yr) reconstructed from the MIROC6 large-ensemble mean SST using the leading two EOF patterns (EOF1 + EOF2); values are plotted as a function of the 44-year trends of PC-1 (x-axis) and PC-2 (y-axis). Grey points represent every 44-year PC-trend pair from individual MIROC6 ensemble members. The black dots show the observed trajectory in Fig, 2a, and the four red points mark the observed positions for 1935–1978, 1951–1994, 1963–2006 and 1979–2022.

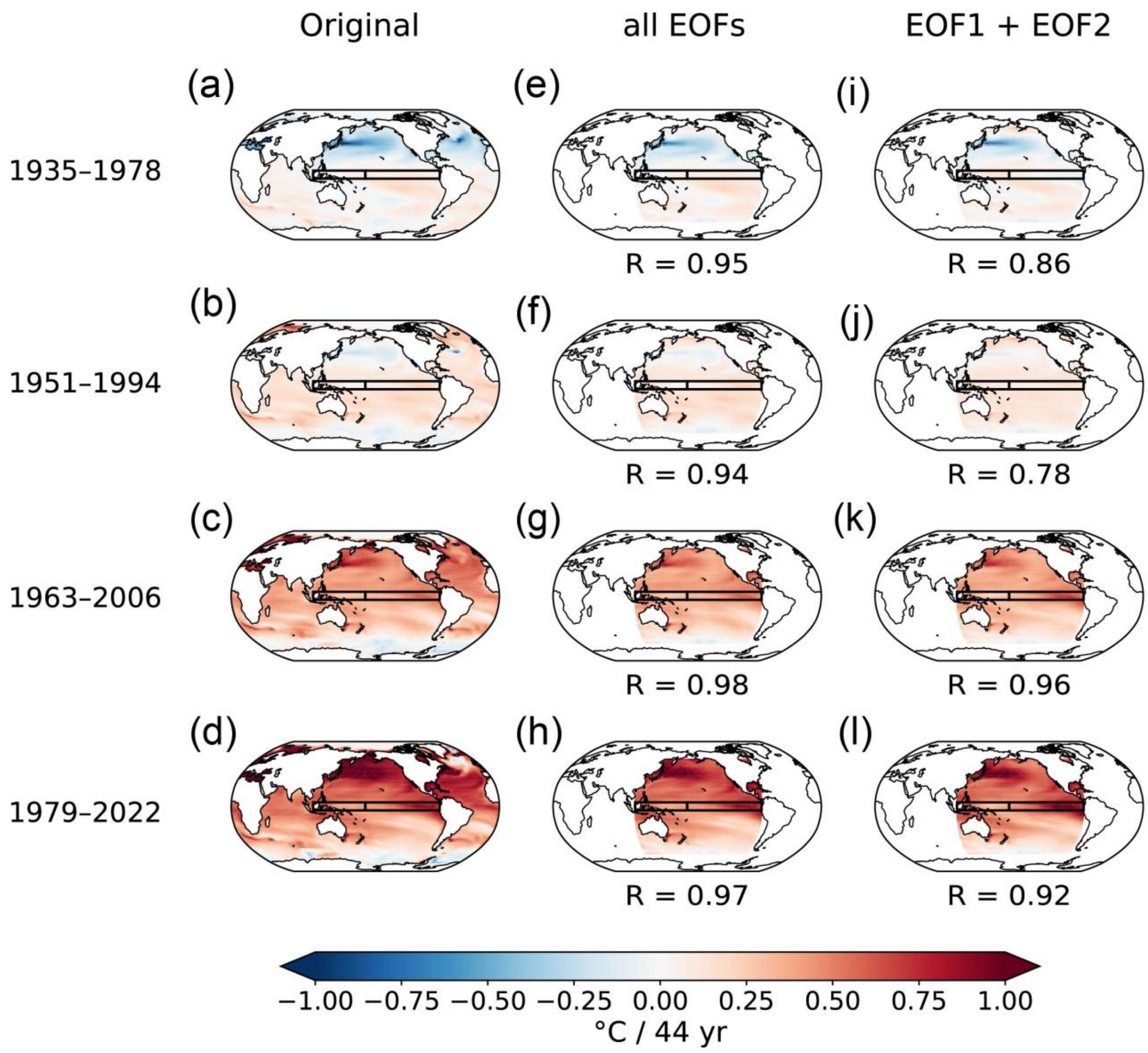

Supplementary Figure 6 | Ensemble-mean and EOF-reconstructed 44-year SST trends in the MIROC6 large ensemble.

Same as Supplementary Figure 3 but for the linear trends in the MIROC6 ensemble mean.

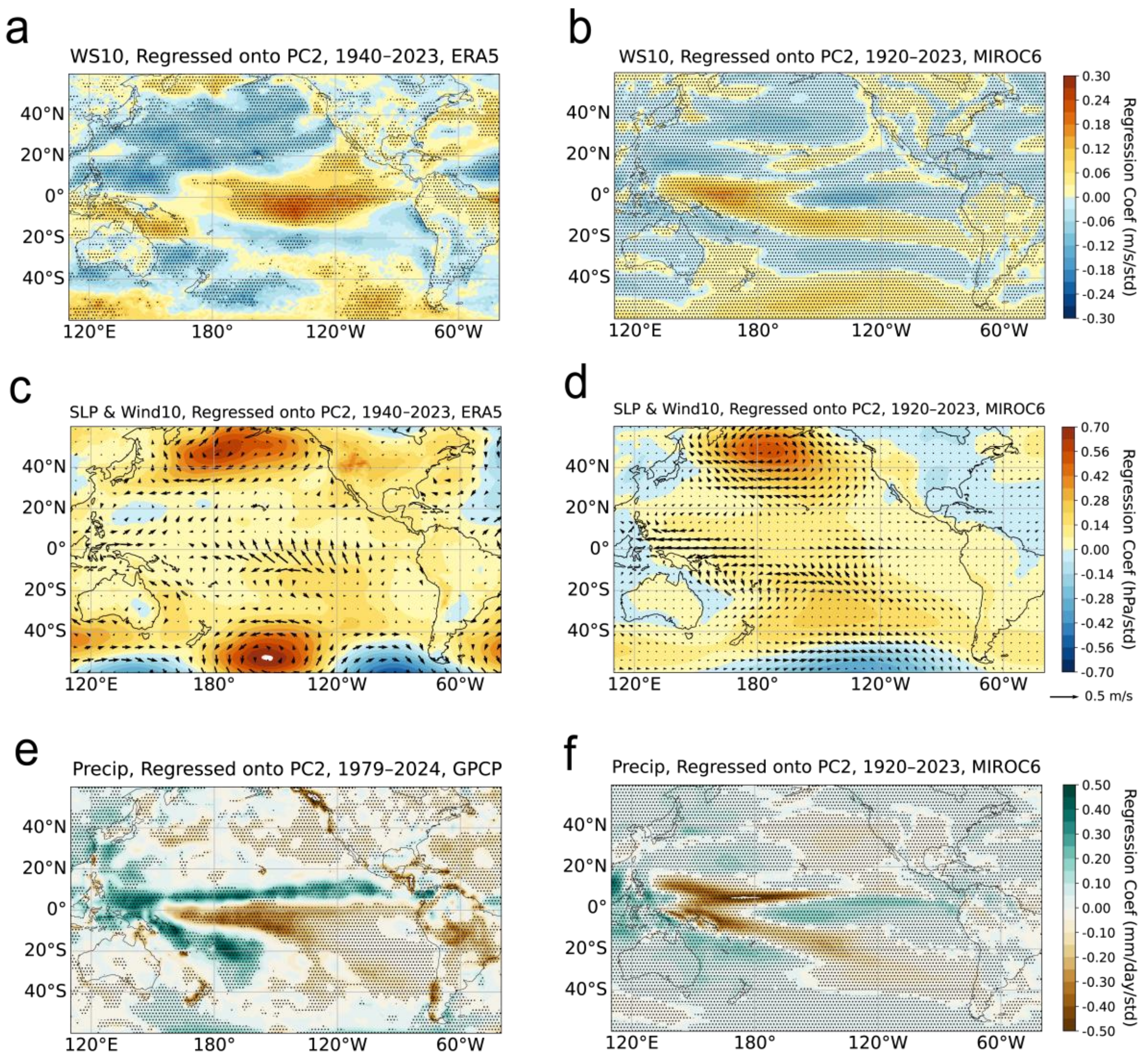

Supplementary Figure 7 | Atmospheric and precipitation patterns associated with PC2.

(a, c, e) Results from reanalysis and observations regressed onto the observed PC2 from ERSSTv5: (a) ERA5 10-m wind speed, (c) ERA5 sea-level pressure (shading) with 10-m wind vectors, and (e) GPCP precipitation. (b, d, f) Results from MIROC6 regressed onto the model's PC2: (b) 10-m wind speed, (d) sea-level pressure with 10-m wind vectors, and (f) precipitation. Stippling indicates significance at the 95% confidence level. Units are m s^{-1} per standard deviation of PC2 for wind speed, hPa per standard deviation of PC2 for sea-level pressure, and mm day^{-1} per standard deviation of PC2 for precipitation.

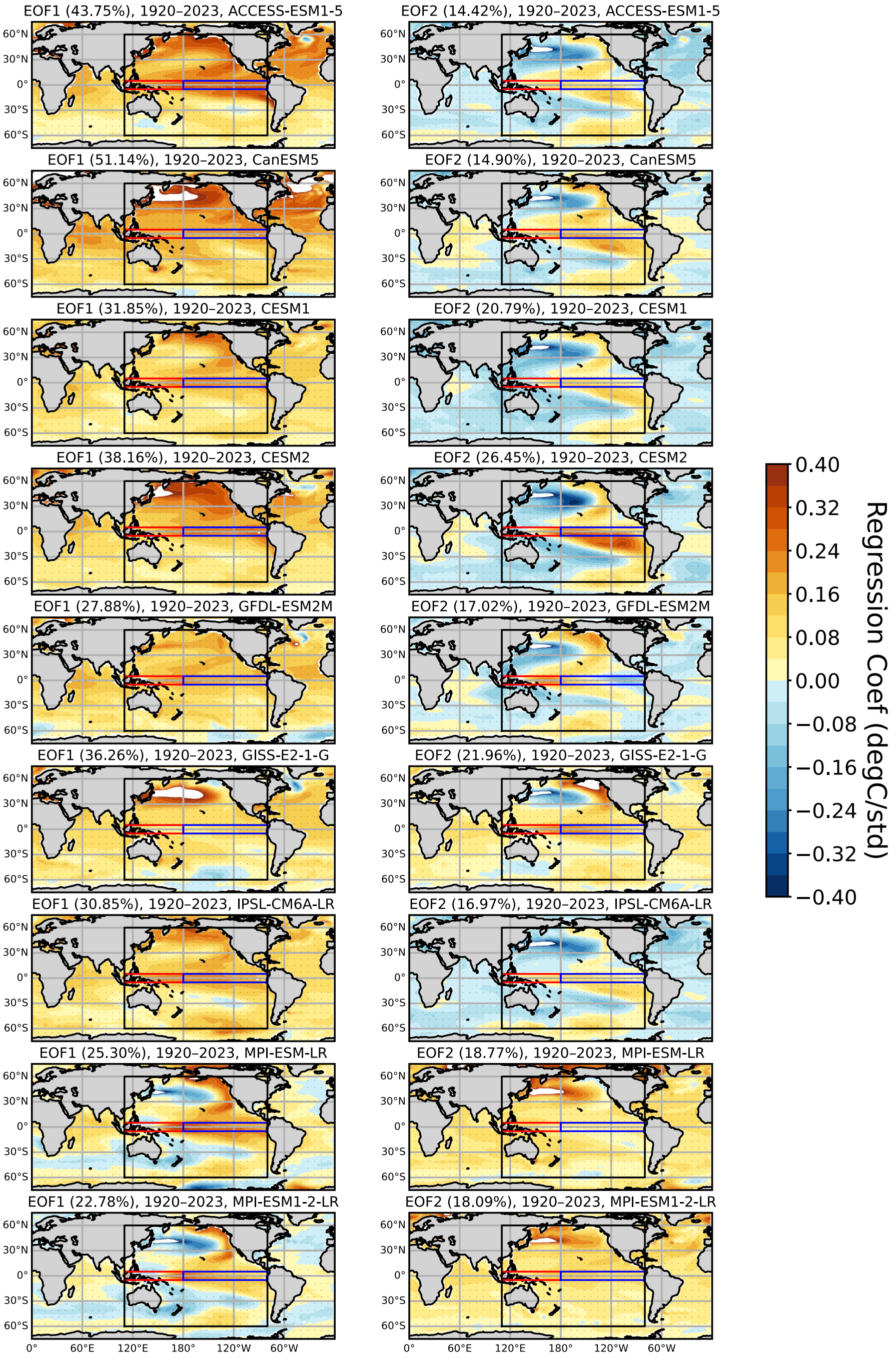

Supplementary Figure 8 | Leading two EOF patterns of low-frequency Pacific SST variability for 1920–2023 in the CMIP large ensembles.

Same as Fig. 3a,b, but for 9 other models: ACCESS-ESM1-5, CanESM5, CESM1, CESM2, GFDL-ESM2M, GISS-E2-1-G, IPSL-CM6A-LR, MPI-ESM-LR, and MPI-ESM1-2-LR.

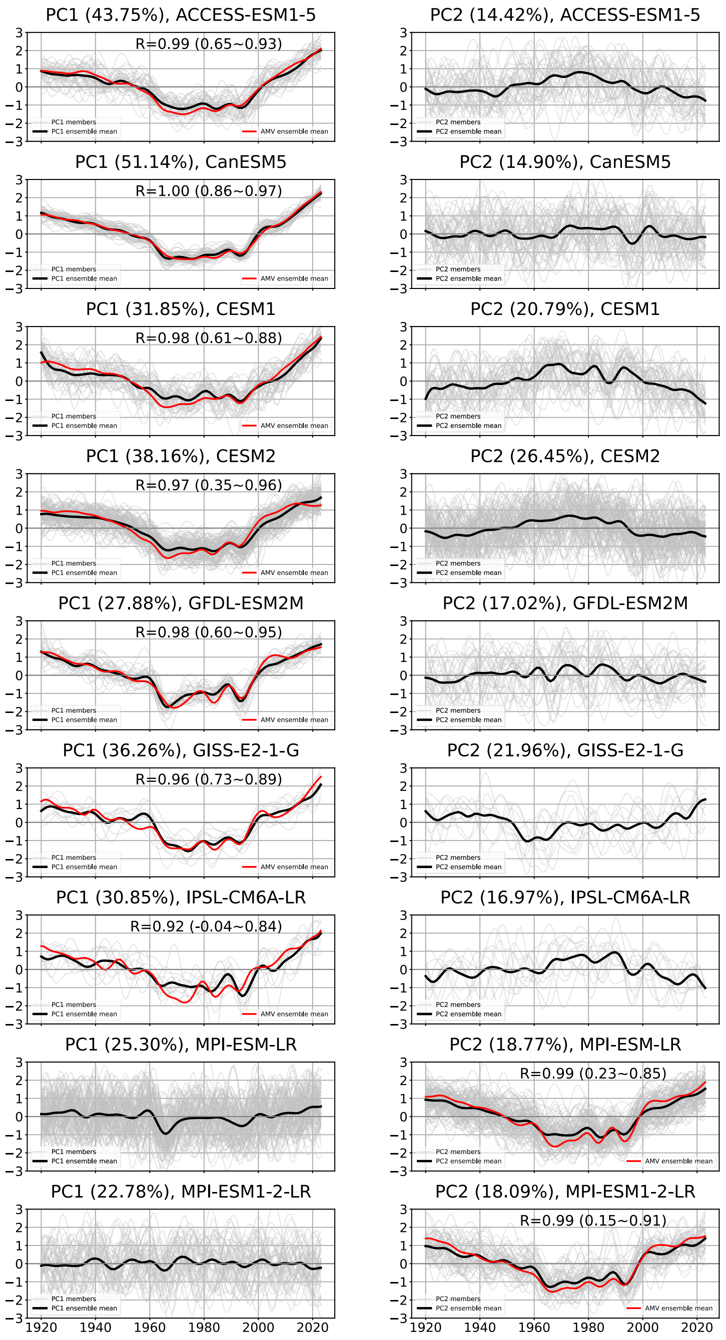

Supplementary Figure 9 | PC time series of the two EOFs in Supplementary Fig. 8.

Same as Fig. 3c,d but for 9 other models: ACCESS-ESM1-5, CanESM5, CESM1, CESM2, GFDL-ESM2M, GISS-E2-1-G, IPSL-CM6A-LR, MPI-ESM-LR, MPI-ESM1-2-LR. For the AMV-related EOF (see Methods), the ensemble-mean time series of the AMV index is overlaid in red with the correlation coefficient shown in the panel.

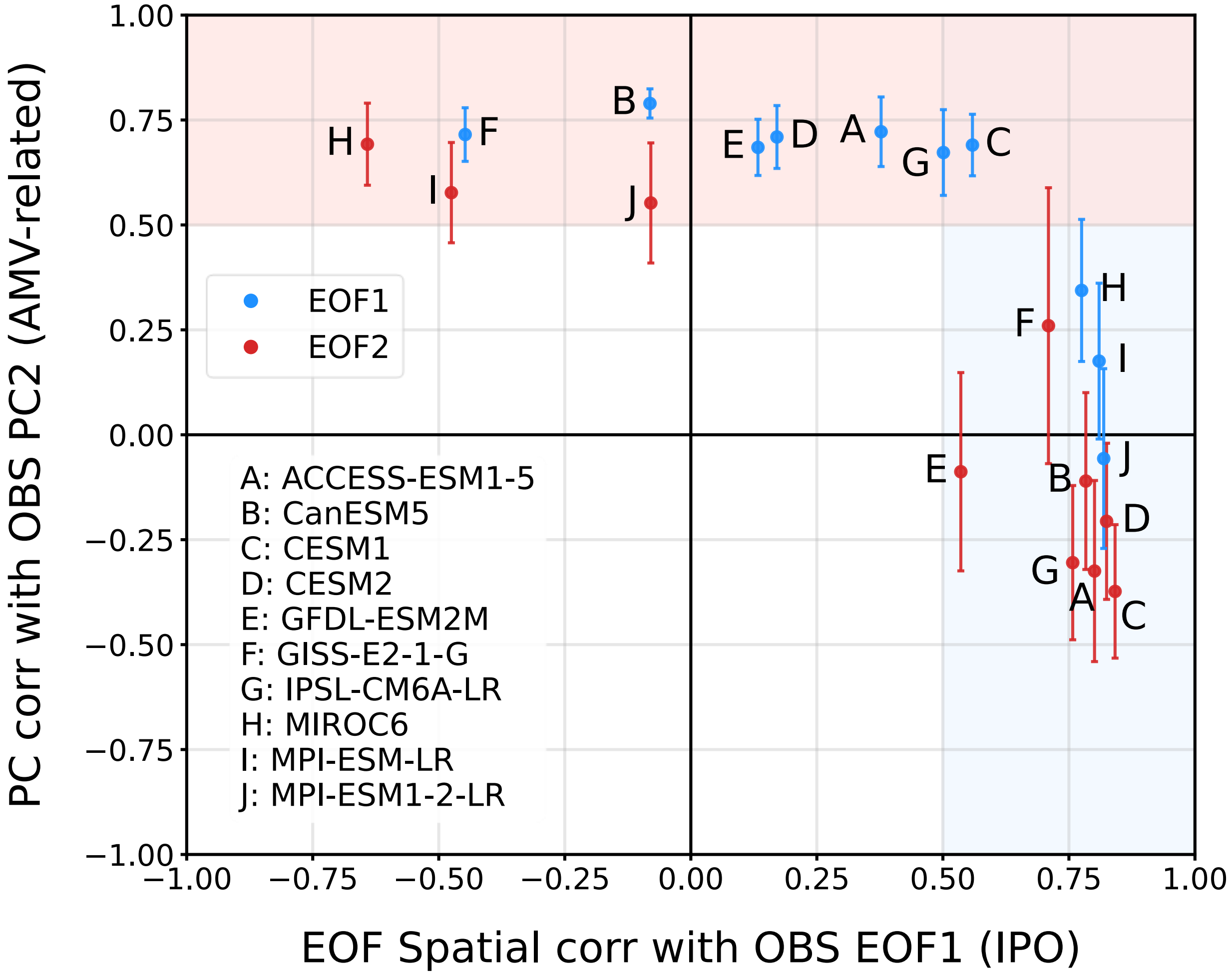

Supplementary Figure 10 | Spatial and temporal correlations between observed and simulated EOFs and PCs.

For each CMIP model, the x-axis shows the spatial correlation between each of its two leading EOF patterns and the observed EOF1 (IPO), and the y-axis shows the temporal correlation between the corresponding PC and the observed PC2 (AMV-related variability). Blue (red) symbols denote EOF1 (EOF2). Dots indicate ensemble-mean correlations, and vertical error bars show the spread across ensemble members. Light-blue shading indicates the spatial correlation with the observed EOF1 exceeding 0.5 and light-red shading represents the temporal correlation with the observed PC2 exceeding 0.5.

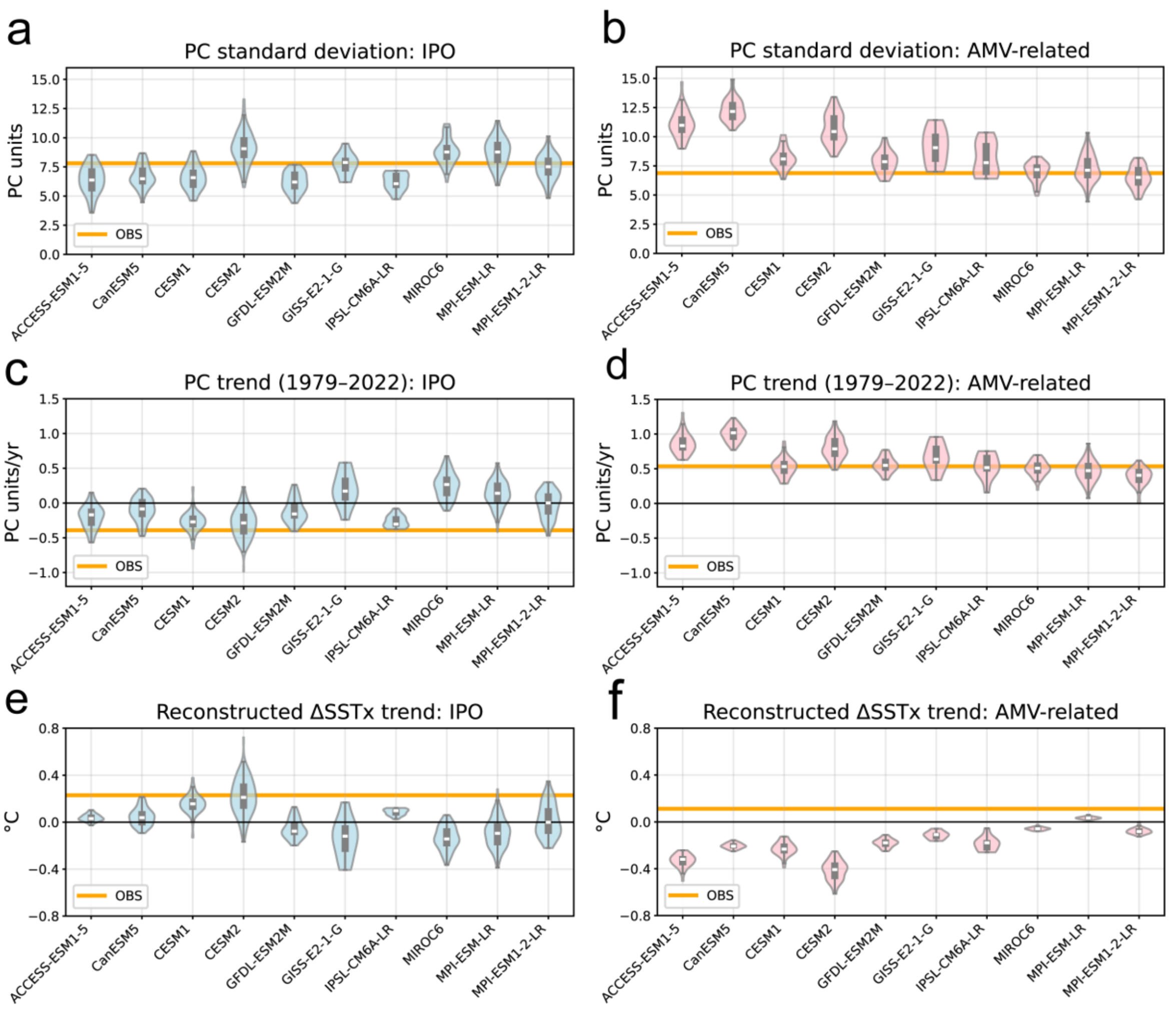

Supplementary Figure 11 | Amplitude and trends of IPO and AMV-related variability in CMIP large ensembles.

Panels (a) and (b) show the standard deviation of the IPO and AMV-related PCs, respectively, computed from the full 1920–2023 time series. Panels (c) and (d) show the 1979–2022 linear trends of the IPO-related and AMV-related PCs. Panels (e) and (f) show the 1979–2022 linear trends of the zonal SST gradient using the reconstructed SST anomalies from the IPO EOF and the AMV-related EOF, respectively. For each model ensemble, the violin plots show the distribution across members, and the boxes and whiskers show the interquartile range and the 5–95th percentiles. The orange horizontal line shows the observed value. For each model, the IPO and AMV-related EOFs and PCs are identified following the classification described in Methods.

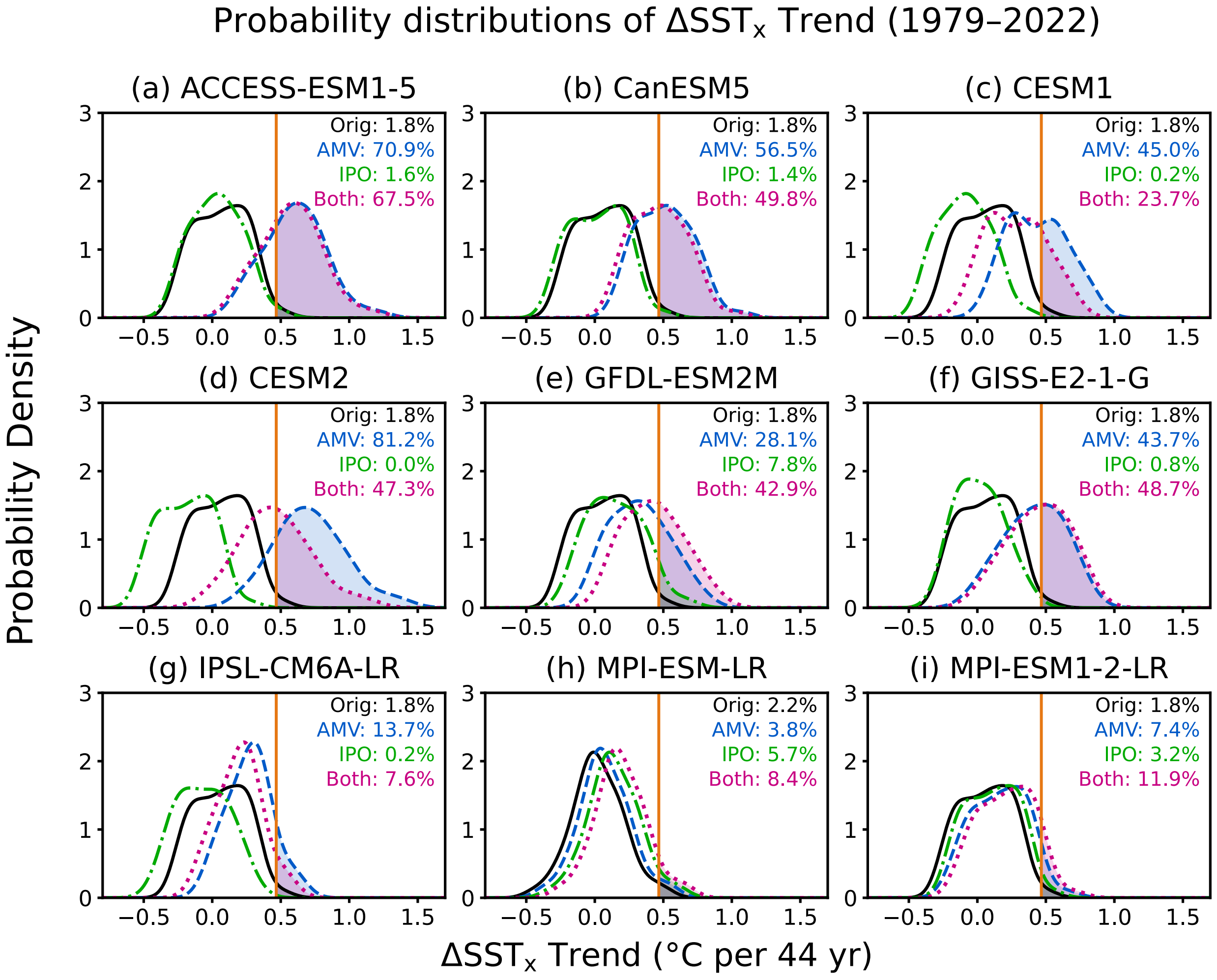

Supplementary Figure 12 | Probability distributions of the trend in the Pacific zonal SST gradient (ΔSST$_x$) for 1979–2022 in CMIP large ensembles.

Same as Fig. 4 but for (a) ACCESS-ESM1-5, (b) CanESM5, (c) CESM1, (d) CESM2, (e) GFDL-ESM2M, (f) GISS-E2-1-G, (g) IPSL-CM6A-LR, (h) MPI-ESM-LR, and (i) MPI-ESM1-2-LR. In each panel, the distributions correspond to (1) Original (black solid), (2) AMV-corrected (blue dashed), (3) IPO-corrected (green dash-dotted), and (4) AMV + IPO-corrected (magenta dotted) curves. The orange vertical line marks the observed trend. The shaded region indicates the fractional area exceeding the observation, and the percentages listed in the upper-right corner give the corresponding exceedance probabilities.

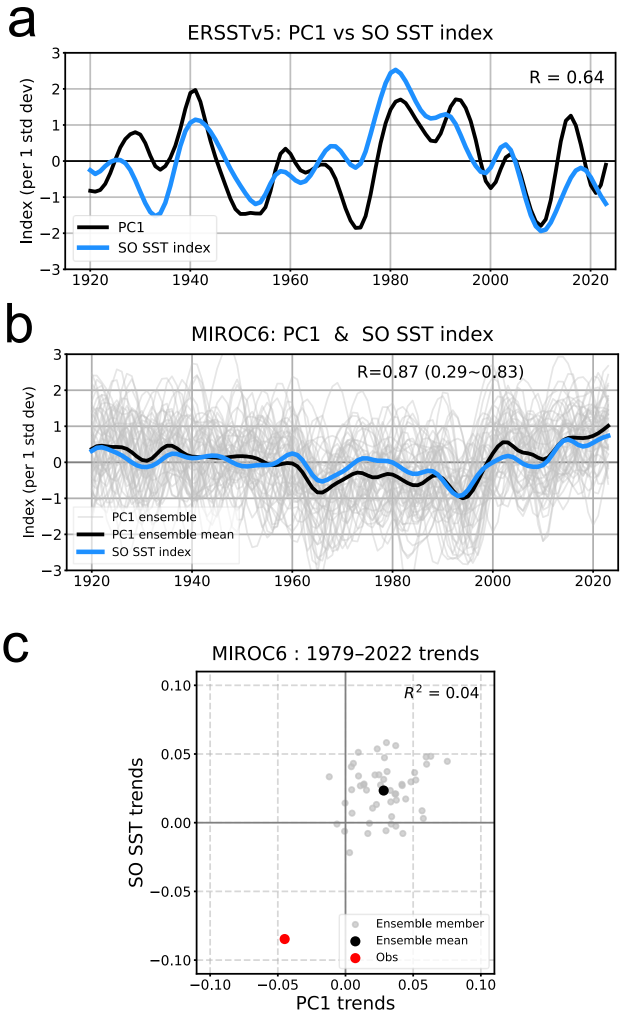

Supplementary Figure 13 | Link between Pacific PC1 and Southern Ocean SST index.

(a) 10-year low-pass filtered Southern Ocean (SO; 75°S–45°S, 110°E–280°E) SST anomaly (black) and PC1 from ERSSTv5 (blue) for 1920–2023. The correlation coefficient is $r = 0.64$. (b) Same as (a), but for the MIROC6 large ensemble. Grey lines show PC1 of individual members; black and blue lines are the ensemble means of PC1 and SO SST index, respectively. The correlation between the ensemble-mean PC1 and the SO SST index $r = 0.87$, with the range computed using individual each member (0.29–0.83). (c) Trends of PC1 and SO SST index over 1979–2022 for each MIROC6 member (grey), ensemble mean (black), and ERSSTv5 (red). The inter-member squared correlation is $r^2 = 0.04$.

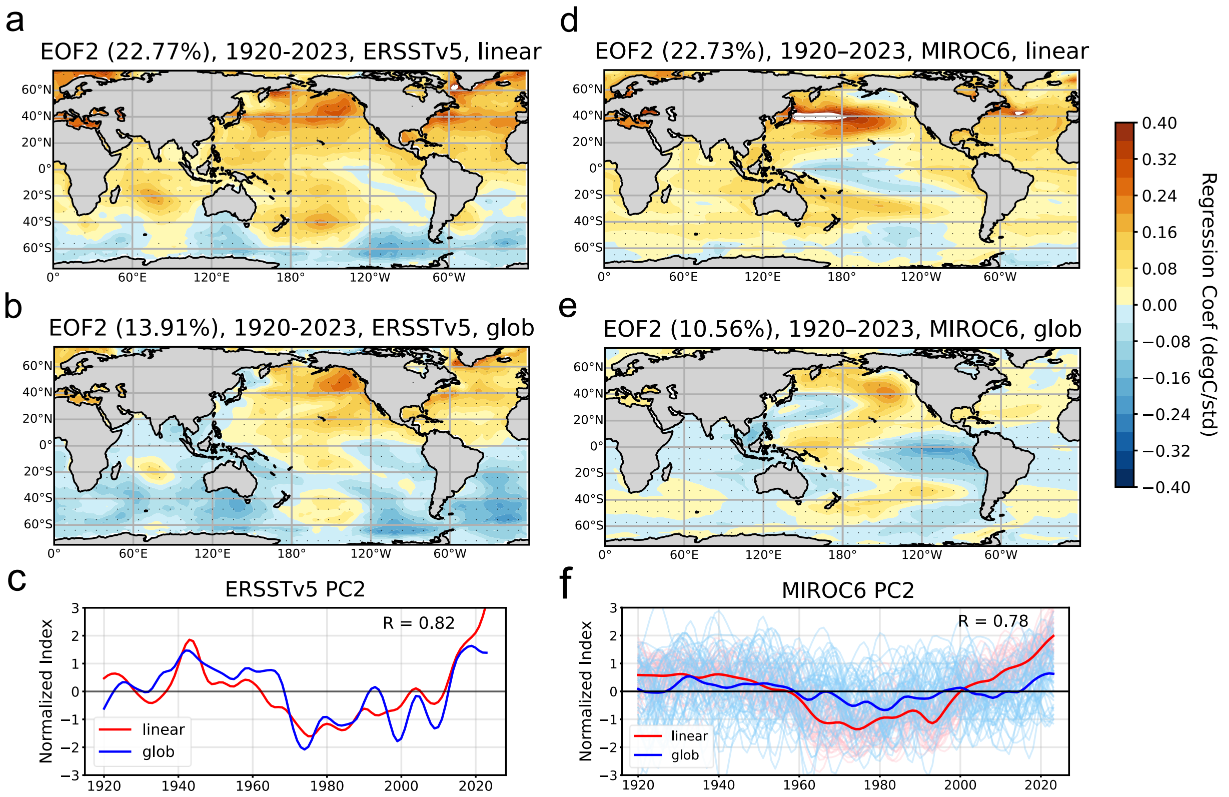

Supplementary Figure 14 | EOF2 patterns and PC2 time series based on the two detrending methods.

(a) Observed EOF2 obtained using linear detrending (replicated from Fig. 1b) and (b) observed EOF2 obtained using global-mean SST–based detrending. (c) Corresponding PC2 time series. (d–f) As in (a–c) but for the MIROC6 large ensemble. See Methods for details of the detrending procedures. Stippling denotes areas with statistically significant values at the 95% confidence level.

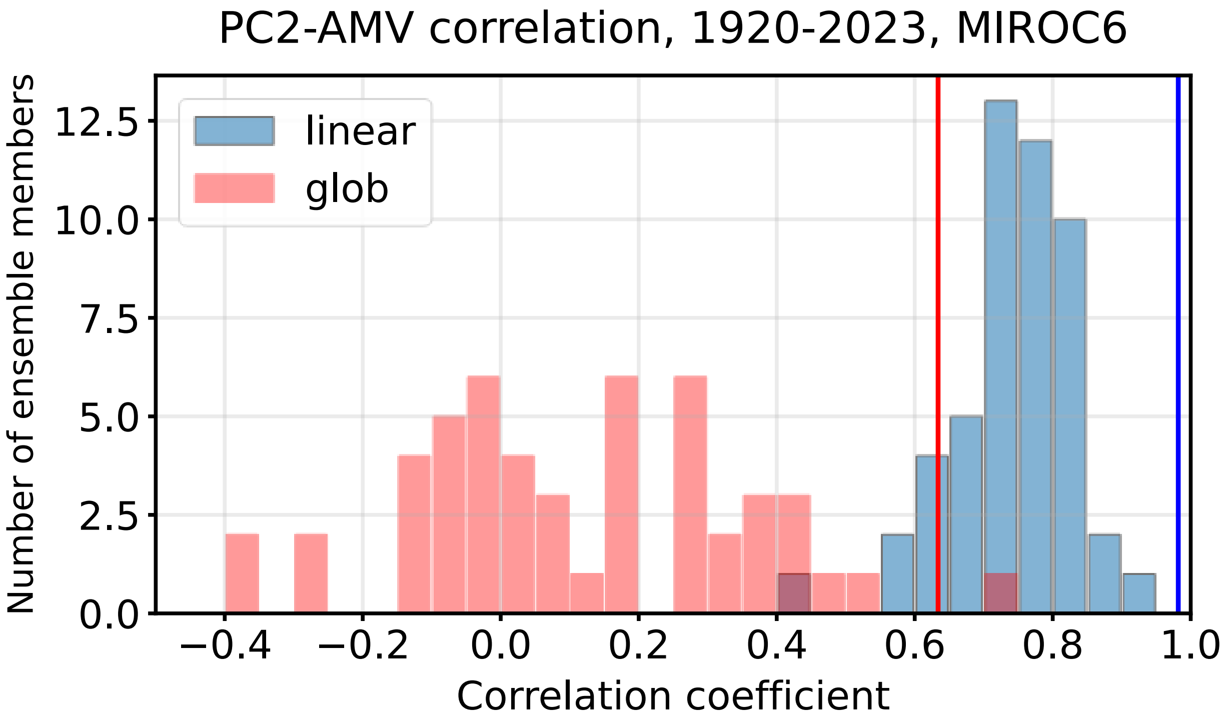

Supplementary Figure 15 | Distribution of the correlation between PC2 and the AMV index across ensemble members in MIROC6.

Histograms show the correlation coefficients between PC2 and the AMV index for individual ensemble members during 1920–2023, obtained using linear detrending (blue) and global-mean SST–based detrending (red). Thick vertical lines denote ensemble-mean correlations. See Methods for details of the detrending procedures and AMV definition.

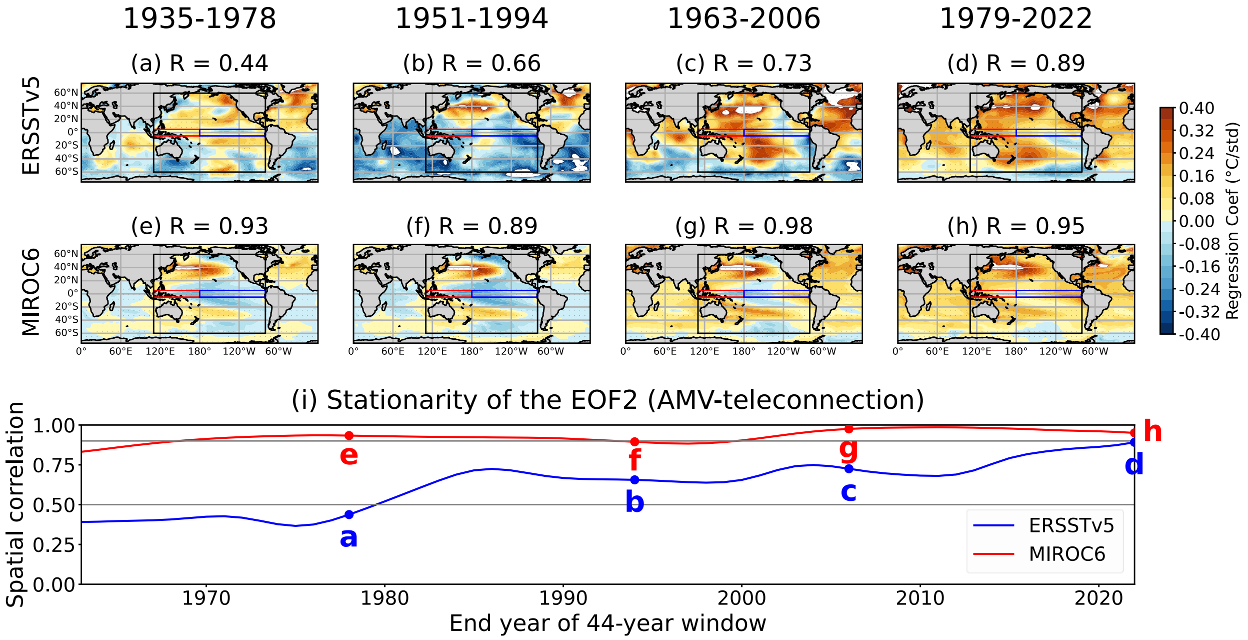

Supplementary Figure 16 | Stationarity of the AMV-related teleconnection pattern in observations and MIROC6.

(a–d) Regression maps of 10-year low-pass filtered SST anomalies onto PC2 from ERSSTv5 for 1935–1978, 1951–1994, 1963–2006, and 1979–2022. (e–h) As in (a–d), but for MIROC6 using concatenated SST anomalies and concatenated PC2 from all ensemble members. Stippling indicates regions significant at the 95% level. R above each panel denotes the spatial correlation with the corresponding full-period (1920–2023) regression map, calculated over the Pacific domain (60°S–60°N, 110°E–280°E). (i) Corresponding 44-year rolling spatial correlations for ERSSTv5 (blue) and MIROC6 (red), with the four periods shown in (a–h) highlighted. See Methods (*"Stationarity of the AMV-related teleconnection"*).

Supplementary Table 1 | The observed trends in the WP SST, EP SST, and ΔSST$_x$ for 1979–2022.

The table compares the contributions of EOF1, EOF2, and their combination (EOF1+EOF2) to the trends (°C per 44 years). The numbers in the parenthesis in the rightmost column represent the fractional percentages against the original SST trends.

ERSSTv5	WP	EP	Zonal gradient WP - EP
EOF1	-0.11	-0.32	0.22 (46%)
EOF2	0.28	0.13	0.15 (32.7%)
EOF1+ EOF2	0.58	0.17	0.40 (86.3%)
All EOFs	0.59	0.12	0.47 (99.9%)
Original SST trend	0.59	0.1	0.47 (100%)

Supplementary Table 2 | ANOVA results for the two leading EOFs in the MIROC6 large ensemble.

Total PC variance ($\sigma^2_{\mathrm{Total}}$) is decomposed into forced ($\sigma^2_{\mathrm{Forced}}$) and internal ($\sigma^2_{\mathrm{Int}}$) components based on the ensemble-mean and deviation PCs, and expressed as fractional contributions (%). Dominant contribution for each EOF is indicated in red. See Methods (*"Attribution of modes of variability in the large ensemble"*).

Mode	$\sigma^2_{\mathrm{Total}}$	$\sigma^2_{\mathrm{Forced}}$	σ^2_{Int}	Contribution of forced response (%)	Contribution of internal variability (%)
EOF1	68.94	14.88	54.07	21.6	**78.4**
EOF2	49.37	33.37	16.00	**67.6**	32.4

Supplementary Text 1 | Large ensemble CMIP5 and CMIP6 simulations.

ACCESS-ESM1-5 LE (The Australian Community Climate and Earth System Simulator Earth System Model, CMIP6 generation) consists of 40 members driven by the CMIP6 historical forcing until 2014 and SSP3-7.0 thereafter. The model resolution is approximately 1.875° × 1.25° in the atmosphere and nominal 1° in the ocean. The data are available through the CMIP6 archive via the Earth System Grid Federation (ESGF).

CanESM5 LE (Canadian Earth System Model version 5, CMIP6 generation) provides 50 members spanning 1850–2014 by the CMIP6 historical forcing and continuing with SSP2-4.5. The model resolution is approximately 2.8° in the atmosphere and nominal 1° in the ocean. The data are distributed by Environment and Climate Change Canada.

CESM1 LE (Community Earth System Model version 1, CMIP5 generation) comprises 40 members driven by the CMIP5 historical forcing up to 2005 and RCP8.5 thereafter. The model resolution is approximately 1° in both the atmosphere and the ocean. The data are provided by the CESM Large Ensemble Community Project.

CESM2 LE (Community Earth System Model version 2, CMIP6 generation) consists of 100 members driven by the CMIP6 historical forcing until 2014 and SSP3-7.0 thereafter. The model resolution is approximately 1.25° × 0.9° in the atmosphere and nominal 1° in the ocean. the ensemble data is available via the NCAR Climate Data Gateway.

GFDL-ESM2M LE comprises 30 members driven by the CMIP5 historical forcing up to 2005 and RCP8.5 thereafter. The model resolution is approximately 2.5° × 2.0° in the atmosphere and nominal 1° in the ocean. The data are provided by NOAA Geophysical Fluid Dynamics Laboratory.

GISS-E2-1-G LE (the NASA Goddard Institute for Space Studies Earth System Model E2.1, CMIP6 generation) contains 10 members that follow the CMIP6 historical protocol up to 2014 and SSP2-4.5 afterwards. The model resolution is approximately 2.5° × 2.0° in the atmosphere and 1.25° × 1° in the ocean. The data are distributed by the NASA Goddard Institute for Space Studies.

IPSL-CM6A-LR LE (Institut Pierre–Simon Laplace model, CMIP6 generation) contains 11 members that follow the CMIP6 historical protocol up to 2014 and SSP2-4.5 afterwards. The model resolution is approximately 2.5° × 1.3° in the atmosphere and nominal 1° in the ocean. The output data are available via the IPSL ESGF node.

MIROC6 LE (Model for Interdisciplinary Research on Climate version 6, CMIP6 generation) consists of 50 members driven by the CMIP6 historical forcing up to 2014 and SSP2-4.5 thereafter. The model resolution is approximately 1.4° in the atmosphere and nominal 1° in the ocean. The ensemble data is available from JAMSTEC upon request.

MPI-ESM-LR LE (Max-Planck-Institute Earth System Model, CMIP5 generation) comprises 100 members driven by the CMIP5 historical forcing up to 2005 and RCP8.5 thereafter. The model resolution is approximately 1.8° in the atmosphere and 1.5° in the ocean. The sea-surface skin temperature fields are taken from the publicly available MPI Grand Ensemble archive.

MPI-ESM1-2-LR LE (Max-Planck-Institute Earth System Model, CMIP6 generation) contains 50 members that follow the CMIP6 historical protocol up to 2014 and SSP2-4.5 afterwards. The model resolution is approximately 1.8° in the atmosphere and 1.5° in the ocean. The data are available through the CMIP6 archive via the ESGF.
