# Supplementary material for: North Atlantic influence reconciling model-observation discrepancy in the tropical Pacific warming pattern
Source: Nat Commun. 2026 May 28;17:6930. doi: 10.1038/s41467-026-73763-0 (PMC13389187; doi:10.1038/s41467-026-73763-0)
Supplement: Supplementary file 2 — Transparent Peer Review file [file 41467_2026_73763_MOESM2_ESM.pdf]

nature portfolio

Peer Review File

North Atlantic influence reconciling model-observation discrepancy in the tropical Pacific warming pattern

Corresponding Author: Ms Yueh-Chi Lin

This file contains all reviewer reports in order by version, followed by all author rebuttals in order by version.

Attachments originally included by the reviewers as part of their assessment can be found at the end of this file.

Version 0:

Reviewer comments:

Reviewer #1

(Remarks to the Author)
The east–west gradient in the tropical Pacific sea surface temperatures (SSTs) is a key determinant of global and regional climate. Observational data show that the zonal SST contrast has intensified over the past few decades, but weakened in majority of climate model simulations. Reconciling these results has been problematic. The authors identified that the simulated SST pattern associated with the Atlantic Multidecadal Variability (AMV) is biased in the tropical Pacific. Correcting the AMV-related SST pattern may partly reconcile the model-observation discrepancy. This paper provided us an interesting view in studying the discrepancy exists between observations and climate models. However, the authors should at times be more cautious in their modeling results, due to the uncertainties in attributing the AMV-related SST variations (mixed internal climate variability and external forcing). My primary comments are as follows.

The issues regarding the two dominant SST patterns in observations --- The authors aimed to quantify the IPO-related and AMV-related SST pattern changes in the tropical Pacific. However, the quantification of the AMV-related variations largely depends on the detrending method. This is particular true under the post-1950 periods where the internal climate variability and changes caused by anthropogenic and natural aerosol forcing mix with each other (Booth et al., 2012; Watanabe & Tatebe, 2019; Qin et al., 2020). The atmospheric teleconnection pathways from the North Atlantic (i.e., AMV) to the Pacific SST variations may be similar regardless whether the AMV is forced or internal (Qin et al., 2024 GRL). Clearly, this does not cleanly remove externally forced decadal changes from the internally generated SST variations in observations by using the EOF analysis (although the linear trend was removed to extract decadal variability), as the externally forced changes are non-linear.

Previous studies used a semi-empirical method that uses a combination of observational data and a large ensemble of coupled climate model simulations to assess the roles of forced and internal variability over the historical period (Steinman et al., 2015 Science) or examine how the internal decadal modes (IPO and AMO, use AMV to refer to the total SST variability while AMO for the natural or internal component) modulated global climate (Dai et al., 2015 Nat Clim Change). This detrending method is chosen over other methods as the multi-model ensemble mean or a single model ensemble mean represent the best estimate of the forced change (Frankcombe et al., 2015; 2018 J. Clim.; Frankignoul et al., 2017 J Clim.).

Furthermore, please note that both internal climate variability and external forcing (e.g., decadal changes in aerosols) contributed to the AMV. If the internal and external AMV are correlated in time, correlative and regression analyses won't be able to distinguish them in North Atlantic SSTs and their remote influences on the tropical Pacific. In other words, AMV-related climate variations may be partly caused by external forcing. The authors need to pay attention to this. Conversely, the IPO index is insensitive to the definition selection (Hua et al., 2018 GRL). Thus, I agree that the EOF1 represents the impact of the IPO on regional SSTs or IPO-related variations in the tropical Pacific or other regions. However, the EOF2 mixes the forced and internal singles, although the temporal and spatial structures resemble the AMV-related variations.

If the authors wanted to examine the effect of the AMO-related variations in the tropical pacific (internal mode, in addition to the IPO) - then they should find another method (e.g., CMIP6-based detrending) to remove the effect of external forcing (which is not a linear trend over the historical period). The results and relative interpretations here do not clearly reflect the effect of the internal and forced changes on Pacific variability. This creates a number of issues, which are evident in figures

1b, 3b, and relevant results.

The AMV's teleconnections arise from both external forcing and internal climate variability (Takahashi and Watanabe, 2016 NCC; Sun et al., 2021 CD; Qin et al., 2024 GRL). While this study does not disentangle their contributions, it highlights discrepancies in model simulations regarding the biases in AMV-related variations in model simulations, especially in the Pacific region. These inconsistencies likely stem from model-dependent wind and SLP anomaly responses to AMV forcing (Sun et al., 2017 NC; 2021 CD; Qin et al., 2024 GRL).

Since the EOF2 represents the AMV-like or AMV-related impacts (as the authors mentioned, regardless whether the AMV is forced or internal), this mode included the forced and internal SST variations. In general, a warm AMO could lead to a cold IPO-like SST pattern in the Pacific Ocean in observations and model simulations (Ruprich-Robert et al., 2017 J. Clim; Meehl et al., 2021 NG). I think the authors could use the North Atlantic pacemaker model simulations (e.g., AMV-related variations on Pacific SST and circulation changes) to examine and explore the model biases reflected in the historical all-forcing model simulations.

(Remarks on code availability)

Reviewer #2

(Remarks to the Author)
Please see attached PDF.

(Remarks on code availability)

Reviewer #3

(Remarks to the Author)
Summary
The study examines potential sources of error in models for incorrectly simulating multi-decadal trends in the zonal gradient of sea surface temperatures (SSTs) in the tropical Pacific. The authors find a relationship between the zonal tropical Pacific SST gradient and multi-decadal variability in Atlantic SSTs in reanalysis products. The relationship indicates that positive values of Atlantic multi-decadal variability (e.g., positive SST anomalies in the Atlantic) yields a strengthened zonal SST gradient across the tropical Pacific (and presumably, the opposite for negative Atlantic SST anomalies). However, climate model simulations show the opposite relationship - i.e., positive Atlantic SST anomalies in the Atlantic over multiple decades leads to a weaker zonal tropical Pacific SST gradient. The authors use some statistical techniques to show that when the Atlantic multi-decadal SST pattern is corrected in the models, the representation of the tropical Pacific zonal SST gradient is improved. However, no dynamical improvements to the models were offered or considered.

Overall Recommendation
The study addresses a very timely and important topic in climate dynamics - the poor simulation of tropical Pacific SST variability in climate models, particularly their fidelity in reproducing observed trends in the field. The authors offered an interesting way of examining this bias in a statistical way and have a "proof of concept" with a few models. However, there are questions as to how generalizable this method is with other models. And certainly, the authors could explore more models from the CMIP6 database to make their point. Even a doubling of the number of models would bolster their case further. Or, in contrast, finding models which actually do simulate the observed trend in the tropical Pacific zonal SST gradient fairly well and contrast those with these models. But, that was not done and leaves a gaping hole in the analysis that is in the scope of the work.

Another issue I have with the manuscript is that there are WAY TOO MANY Supplemental Figures to consider for the analyses. Supplemental Figures should only be used to further support some arguments in the paper, not be relied on to make actual arguments or support of the hypotheses. However, this is exactly what the authors do - e.g., the "Multi-model analysis" section of the paper refers only to Supplemental Figures (and many of them!) to present results. This over-reliance on Supplemental Figures is a classic signal is that the study is not well-equipped for a short format journal like this. And, with my suggested addition of examining more models and contrasting "poor-performing" versus "better" models, the main sections of the paper would need to be expanded even further.

For these reasons, I am recommending that the paper be rejected for publication in Nature Communications and instead be resubmitted to another longer-format journal for publication.

(Remarks on code availability)
There was no code available for review.

Version 2:

Reviewer comments:

Reviewer #1

(Remarks to the Author)
The authors have addressed all of my concerns and suggestions in the revised manuscript.

(Remarks on code availability)

Reviewer #2

(Remarks to the Author)
Please see pdf.

(Remarks on code availability)

Reviewer #3

(Remarks to the Author)
Please see attached review.

(Remarks on code availability)

Version 3:

Reviewer comments:

Reviewer #3

(Remarks to the Author)
This is my third review of this paper. The authors have made substantial and thoughtful revisions based on the numerous comments. The manuscript is now clearer in message and better supported analytically. The central hypothesis, that a biased AMV–tropical Pacific teleconnection pattern is a major contributor to the model vs. observation discrepancy in recent tropical Pacific sea surface temperature (SST) gradient trends, is more convincingly demonstrated. The authors also nicely addressed my major concerns on sample size for statistical testing and also the idea of stationarity in the relationship. I still find the paper interesting and scientifically sound. Therefore, I recommend the paper be accepted for publication.

(Remarks on code availability)

Open Access This Peer Review File is licensed under a Creative Commons Attribution 4.0 International License, which permits use, sharing, adaptation, distribution and reproduction in any medium or format, as long as you give appropriate credit to the original author(s) and the source, provide a link to the Creative Commons license, and indicate if changes were made.
In cases where reviewers are anonymous, credit should be given to 'Anonymous Referee' and the source.
The images or other third party material in this Peer Review File are included in the article's Creative Commons license, unless indicated otherwise in a credit line to the material. If material is not included in the article's Creative Commons license and your intended use is not permitted by statutory regulation or exceeds the permitted use, you will need to obtain permission directly from the copyright holder.
To view a copy of this license, visit https://creativecommons.org/licenses/by/4.0/

NCOMMS-25-54711-T

North Atlantic influence reconciling model-observation discrepancy in the tropical Pacific warming pattern

by Lin and Watanabe

Reply to the comments by Reviewer #1

We thank the reviewer for carefully reading our manuscript and providing constructive comments. We addressed all the comments and revised the manuscript accordingly.

Please see our point-by-point responses below.

The east–west gradient in the tropical Pacific sea surface temperatures (SSTs) is a key determinant of global and regional climate. Observational data show that the zonal SST contrast has intensified over the past few decades, but weakened in majority of climate model simulations. Reconciling these results has been problematic. The authors identified that the simulated SST pattern associated with the Atlantic Multidecadal Variability (AMV) is biased in the tropical Pacific. Correcting the AMV-related SST pattern may partly reconcile the model-observation discrepancy. This paper provided us an interesting view in studying the discrepancy exists between observations and climate models. However, the authors should at times be more cautious in their modeling results, due to the uncertainties in attributing the AMV-related SST variations (mixed internal climate variability and external forcing). My primary comments are as follows.

We thank the positive evaluation to our work. We explain our approach about attribution in our reply below.

The issues regarding the two dominant SST patterns in observations --- The authors aimed to quantify the IPO-related and AMV-related SST pattern changes in the tropical Pacific. However, the quantification of the AMV-related variations largely depends on the detrending method. This is particular true under the post-1950 periods where the internal climate variability and changes caused by anthropogenic and natural aerosol forcing mix with each other (Booth et al., 2012; Watanabe & Tatebe, 2019; Qin et al., 2020).

We acknowledge that the AMV-related variability may be sensitive to the detrending method. To address your concern, we repeated the EOF analysis using a different detrending method (called 'glob' against our method 'linear') in which SST anomalies regressed onto the global-mean SST time series (60°S–60°N) have been removed in advance. In observations, the AMV-related variability represented as the EOF2 was similar with the two detrending procedures. The tropical Pacific zonal SST-gradient trend for 1979–2022 remains nearly unchanged (0.43 vs 0.42 °C per 44 yr) (Supplementary Fig. 14a,b). The associated PC2 time series also exhibits coherent multidecadal variability under both detrending methods (Supplementary Fig. 14c), indicating that the AMV-related EOF2 structure identified in the Pacific is not an artifact of a choice of the detrending method. However, the EOF2 pattern in MIROC6 with the 'glob' detrending was found very different from the EOF2 with the 'linear' detrending, and furthermore, the ensemble-mean shows a much weak signal (Supplementary Fig. 14d-f). This indicates that the AMV-related variability in the model (Fig. 2b) occurs largely due to externally forced response (see our reply to your next comment) and thus tightly coupled with the global-mean SST variability, which is also suggested to include a bias. This is further supported by Supplementary Fig. 15. Under 'linear' detrending, PC2–AMV correlations are consistently high across ensemble members. Under the 'glob' detrending, the correlations shift to lower values and show a much broader spread, and the ensemble-mean correlation decreases from 0.98 to 0.63. This suggests that the 'glob' detrending removes part of the variability relevant to the AMV–Pacific linkage in MIROC6. Therefore, we have not changed our detrending method.

The above results do not degrade our conclusions, but worth mentioning. We have added a new section 'Sensitivity to detrending methodology' in the Methods and Supplementary Figs. 14 and 15 in explaining that the model biases in the AMV-related variability has a link to the global-mean SST variability and therefore we adopted the linear detrending method for our analysis. These explanation and figure have also been referred to in the main text at L.136-141.

The atmospheric teleconnection pathways from the North Atlantic (i.e., AMV) to the Pacific SST variations may be similar regardless whether the AMV is forced or internal (Qin et al., 2024 GRL). Clearly, this does not cleanly remove externally forced decadal changes from the internally generated SST variations in observations by using the EOF analysis (although the linear trend was removed to extract decadal variability), as the externally forced changes are non-linear. Previous studies used a semi-empirical method that uses a combination of

observational data and a large ensemble of coupled climate model simulations to assess the roles of forced and internal variability over the historical period (Steinman et al., 2015 Science) or examine how the internal decadal modes (IPO and AMO, use AMV to refer to the total SST variability while AMO for the natural or internal component) modulated global climate (Dai et al., 2015 Nat Clim Change). This detrending method is chosen over other methods as the multi-model ensemble mean or a single model ensemble mean represent the best estimate of the forced change (Frankcombe et al., 2015; 2018 J. Clim.; Frankignoul et al., 2017 J Clim.).
Furthermore, please note that both internal climate variability and external forcing (e.g., decadal changes in aerosols) contributed to the AMV. If the internal and external AMV are correlated in time, correlative and regression analyses won't be able to distinguish them in North Atlantic SSTs and their remote influences on the tropical Pacific. In other words, AMV-related climate variations may be partly caused by external forcing. The authors need to pay attention to this. Conversely, the IPO index is insensitive to the definition selection (Hua et al., 2018 GRL). Thus, I agree that the EOF1 represents the impact of the IPO on regional SSTs or IPO-related variations in the tropical Pacific or other regions. However, the EOF2 mixes the forced and internal singles, although the temporal and spatial structures resemble the AMV-related variations.

Thank you for the comment. First, we would like to make ourselves clear that we did not intend to remove externally forced decadal–multidecadal changes in observations and models. Our primary purpose was to identify possible model biases in representing the tropical Pacific SST trend pattern no matter what caused them. Yet, we agree that the attribution of the AMV-related variability to internal mode of variability (i.e., AMO) and externally forced response is important for how to interpret our results.

Given possibility that the forced responses in climate models are biased (Wills et al. 2022, Seager et al. 2022, Watanabe et al. 2024, Simpson et al. 2025, and others), we could not take a methodology that combines observations and large-ensemble simulations for separating the internal variability and forced responses in observational records. Therefore, we did not discuss their roles in observed EOFs in this paper. On the other hand, we could separate internally generated and externally forced components in the large-ensemble itself, following a conventional definition that the ensemble mean represents the forced component and ensemble deviations represent internally generated component. The results of the EOF analyses to those components in MIROC6 have newly been

presented in Supplementary Fig. 4. The EOF1 to the forced component, accounting for about 90% of the total variance, shows warming in the North Pacific and Atlantic and a weakened zonal SST contrast in the equatorial Pacific. This pattern resembles the AMV-related pattern shown in Fig. 3b, and the PC time series are similarly indicating the multidecadal signal. In contrast, the EOF1 to the internal component, accounting for about 38% of the total variance, clearly exhibits the IPO pattern consistent with Fig. 3a. This result supports our arguments that the IPO (Fig. 3a) is mainly internally generated whereas the AMV-related variability (Fig. 3b) is largely induced by external forcing.

To further quantify the forced versus internally generated components in the model, we have applied the "analysis of variance" (ANOVA) (Rowell 1998) to the EOF results of the MIROC6 large ensemble shown in Fig. 3. In ANOVA, the PC time series is decomposed into the ensemble mean and deviations from the ensemble mean. The variances using the respective components, denoted as $\sigma^2_{\mathrm{Forced}}$ and σ^2_{Int}, are then divided by the variance of the raw PC time series, $\sigma^2_{\mathrm{Total}}$, leading to the fractional contribution of the forced response and internal variability. The results presented below in **Supplementary Table 2,** consistently show that the EOF2 (AMV-related variability) is predominantly forced (67.6%) whereas the EOF1 (IPO) is mostly induced by internal variability (78.4%). We have explained these additional results in the revised text at L.114-117 & L138-141 and a new section "Attribution of modes of variability in the large ensemble" in the Methods.

Supplementary Table 2 | ANOVA results for the two leading EOFs in the MIROC6 large ensemble. Total PC variance ($\sigma^2_{\mathrm{Total}}$) is decomposed into forced ($\sigma^2_{\mathrm{Forced}}$) and internal ($\sigma^2_{\mathrm{Int}}$) components based on the ensemble-mean and deviation PCs. Dominant contribution for each EOF is indicated in red.

Mode	$\sigma^2_{\mathrm{Total}}$	$\sigma^2_{\mathrm{Forced}}$	σ^2_{Int}	Contribution of forced response (%)	Contribution of internal variability (%)
EOF1	68.94	14.88	54.07	21.6	78.4
EOF2	49.37	33.37	16.00	67.6	32.4

If the authors wanted to examine the effect of the AMO-related variations in the tropical pacific (internal mode, in addition to the IPO) - then they should find another method (e.g., CMIP6-based detrending) to remove the effect of external forcing (which is not a linear trend over the historical period). The results and relative interpretations here do not clearly reflect the effect of the internal and forced changes on Pacific variability. This creates a number of issues, which are evident in figures 1b, 3b, and relevant results.

As explained above, we did not aim to examine the effect of the internal Atlantic SST variability, AMO, on the tropical Pacific. Please see our reply to your previous comments, too.

The AMV's teleconnections arise from both external forcing and internal climate variability (Takahashi and Watanabe, 2016 NCC; Sun et al., 2021 CD; Qin et al., 2024 GRL). While this study does not disentangle their contributions, it highlights discrepancies in model simulations regarding the biases in AMV-related variations in model simulations, especially in the Pacific region. These inconsistencies likely stem from model-dependent wind and SLP anomaly responses to AMV forcing (Sun et al., 2017 NC; 2021 CD; Qin et al., 2024 GRL).

We appreciate your correct understanding that our main conclusions point out possible biases in the AMV-related pattern in the Pacific in climate models, which then reconcile part of the model-observation discrepancy in the Pacific zonal SST gradient trend. The conclusions do not, in principle, require the attribution of the AMV, but we have argued in the revised text that biases in large ensembles arise mainly from failure in representing external forced response associated with the AMV.

Since the EOF2 represents the AMV-like or AMV-related impacts (as the authors mentioned, regardless whether the AMV is forced or internal), this mode included the forced and internal SST variations. In general, a warm AMO could lead to a cold IPO-like SST pattern in the Pacific Ocean in observations and model simulations (Ruprich-Robert et al., 2017 J. Clim; Meehl et al., 2021 NG).

Although around 70% of the model's EOF is explained by the forced variations (**Supplementary Table 2**), a set of the North Atlantic warming and the equatorial Pacific cooling might come from internal variability. This out-of-phase relationship is clearer when we repeat the EOF analysis to the ensemble deviations. The EOF1, which represents the IPO, shows a warm IPO pattern accompanied

by a cooling in the North Atlantic (**Supplementary Fig. 4b**), consistent with the reviewer's point.

I think the authors could use the North Atlantic pacemaker model simulations (e.g., AMV-related variations on Pacific SST and circulation changes) to examine and explore the model biases reflected in the historical all-forcing model simulations.

Thank you for the suggestion. However, our results suggest that the North Atlantic pacemaker experiments will not resolve the problem because the model bias appears in the teleconnection pattern in the Pacific but not in the time evolution of the AMV itself as seen in the similarity in PC time series (Figs. 1d and 3d, the correlation between the observed PC and the ensemble-mean PC is $r = 0.83$). A possibility causing the teleconnection pattern bias is errors in the climatological mean states in the tropical Pacific, so flux-adjusted pacemaker experiments may be promising to verify our arguments. Conducting such experiments requires is beyond the scope of this study but should be a subsequent work. We have now briefly discussed these possible numerical experiments to step further forward in in the closing section.

NCOMMS-25-54711-T

North Atlantic influence reconciling model-observation discrepancy in the tropical Pacific warming pattern

by Lin and Watanabe

Reply to the comments by Reviewer #2

We thank the reviewer for providing positive comments and clear guidance to improve our paper. We have revised the manuscript according to your suggestion and comments. Please see our point-to-point reply below.

This manuscript investigates the strengthening in the tropical Pacific SST gradient in observations and seeks to explain why climate model simulation have a weaken SST gradient. The authors attribute model biases in the Atlantic multidecadal variability as a key reason to explain why models fail to capture the observed SST strengthening. The authors have carried out an interesting analysis and have generated a large number of figures to support their findings. I note that Professor Watanabe is a world leader in this area of research and, as such, is an authority on this topic. I anticipate the manuscript will be suitable for publication in Nature communications. I have two main areas which impacted by ability to follow the analysis. While reading the manuscript this became a distraction to my enjoyment of the article, so for this reason I as suggesting major revisions. I noticed quite of lot of small text changes that I could suggest, but I will include these in the second round of review, as I don't want them to distract from the science. Nature communications is a journal with a quick turn around so I don't expect this will delay the process.

Thank you very much for your encouraging comment.

Major Comments

1. The IPO is assumed to be the prominent multi-decadal influence of the tropical Pacific gradient. The PDO is also known to impact the the gradient as well. Could I suggest you justify why the IPO was considered but not the PDO as well please? I realise the PDO is more constrained to the NH, but it still impacts the tropical Pacific SSTs. Perhaps it is as simple as the IPO and PDO are phase locked but I would like to hear the authors thoughts and justifations on

this please.

Thank you for the comment and question. We acknowledge the literature refers to both IPO and PDO as a dominant decadal mode of variability in the Pacific. As you point out, the definition of the PDO is confined to the North Pacific (110°E–280°E, 20°–70°N) whereas the IPO is defined in the entire Pacific (110°E–280°E, 60°S–60°N). As we attempted to extract dominant patterns of SST variability that explain the tropical Pacific zonal SST gradient change (ΔSST_x) using the EOFs to the entire Pacific basin, it was natural to regard the EOF1 as the IPO, supported by a high correlation between the PC1 and a conventional IPO index. However, given that the IPO and PDO are also highly correlated (e.g., Newman et al. 2016), the influence of the IPO on ΔSST_x would include the effect of the PDO. Indeed, no PDO-like EOF appeared in our analysis, indicating that the PDO being largely embedded in the EOF1. A similar argument applies to the so-called tropical Pacific decadal variability, TPDV (Capotondi et al. 2023). We have briefly mentioned these modes of variability and a relationship with the EOF1 at L.22–27.

2. Reconstruction tests: I like the idea and I applaud the effort, but I don't understand what has been done so I am struggling to know if I agree with your assessment or not. In L184-188 the method is described, but not in enough detail so that I know what has been done. Could you comment on if this is a common method (I am not familiar with it), there are no references in this section it might help to add some, and I suggest you expand the methods section to explain this in more detail. Could you also clearly say this is a statistical test of the model data and that simulations were not repeated with altered model set-up. This was my initial thought until I realised you have done it for the other models too.

Thank you for the comment. First, the reconstruction test is based on a commonly used EOF-based statistical approach that has been adopted in previous studies (e.g., Dai et al., 2015, Nature Climate Change, https://doi.org/10.1038/nclimate2605). To increase readability, we have expanded the Methods by adding a new subsection "Reconstruction test" with equations, a brief step-by-step description, and explicit statement that this "what-if" test was applied to the model outputs without further model simulations. Here we repeat the description in the revised Methods.

Reconstruction tests.

An EOF-based reconstruction of tropical Pacific SST anomalies is used to examine how the potential

errors in IPO and AMV-related variability influence the ΔSST_x trend for 1979–2022. For large-ensemble model simulations, annual SST anomalies for 1920–2023 are decomposed into a centennial-scale linear trend and a set of EOFs that are computed from the concatenated ensemble × time dimension. A baseline reconstructed SST field, $SST_{\text{recon}}(x,t)$, is expressed as

$$SST_{\text{recon}}(x,t) = SST_{\text{cent}}(x,t) + \sum_{k=1}^{N} PC_k(t) EOF_k(x) \quad ,$$

where $SST_{\text{cent}}(x,t)$ is the local linear trend for 1920–2023, $EOF_k(x)$ is the k-th EOF pattern, and $PC_k(t)$ is its associated time series. The number of EOF is truncated at N=104, which corresponds to the maximum degrees of the freedom in observations, i.e., 104 years for 1920–2023. For each of 10 LEs, the leading two EOFs are identified to represent IPO and the AMV-related variability like observations. Their patterns, denoted as $EOF_{\text{IPO}}(x)$ and $EOF_{\text{AMV}}(x)$, and associated PC time series, $PC_{\text{IPO}}(t)$ and $PC_{\text{AMV}}(t)$ are modified in the reconstruction test. A correction to the AMV-related pattern replaces $EOF_{\text{AMV}}(x)$ in a model with the observed pattern $\text{EOF}_{\text{AMV}}^{\text{obs}}(x)$ while keeping $PC_{\text{AMV}}(t)$ unchanged:

$$\text{SST}_{\text{mod,AMV}}(x,t) = \text{SST}_{\text{recon}}(x,t) - PC_{\text{AMV}}(t)\,\text{EOF}_{\text{AMV}}(x) + PC_{\text{AMV}}(t)\,\text{EOF}_{\text{AMV}}^{\text{obs}}(x)$$

On the other hand, the IPO correction removes the ensemble-mean (forced) component in $PC_{\text{IPO}}(t)$, denoted as $\langle PC_{\text{IPO}}(t)\rangle$ and therefore the corrected reconstruction will be

$$\text{SST}_{\text{mod,IPO}}(x,t) = \text{SST}_{\text{orig}}(x,t) - PC_{\text{IPO}}(t)\,\text{EOF}_{\text{IPO}}(x) + PC_{\text{IPO}}^{\text{int}}(t)\,\text{EOF}_{\text{IPO}}(x) \quad ,$$

where

$$PC_{\text{IPO}}^{\text{int}}(t) = PC_{\text{IPO}}(t) - \langle PC_{\text{IPO}}(t)\rangle$$

represents internally generated component of IPO. The combined reconstruction can be made by using both corrections. These EOF-based reconstructions provide a "what-if" test for the ΔSST_x trend in climate models when biases in the IPO and AMV-related variability in the models are eliminated and can be done without performing additional simulations.

Minor Comments

1. In Fig. 2a, the colour scale in the background represents the trends. Could you comment on the anticorrelated nature of the two PCs please? (i.e. why a PC1 of .1 related to a PC2 of -.1). I want to better understand the inherent structure behind the dots.

By definition, EOF1 and EOF2 are orthogonal and their associated PCs are uncorrelated at zero lag. Therefore, the shading in Fig. 2a does not show a relationship between the PCs but

demonstrates dependence of the zonal SST gradient trend on the trends of the PCs. This dependence was calculated based on SST anomalies reconstructed using the two EOF patterns but with varying values in the PC trends, so we have cited Methods when explaining Fig. 2a. We have also rewritten the paragraph at L.84–89 to help readers understand what the figure represents.

2. Fig 2. I like it. It took me a while to 'get it' but it is interesting. Do you think we can say anything about the forced response by the fact the shape is not a closed circle (i.e. a lifecycle of a mode of variability) and that the latter 10 time windows or so move into a new part of the phase space?

Thank you for pointing out an interesting feature in Fig. 2a. We are aware that the trajectory was like a limit cycle during the early period but moved away toward a more positive PC2 trend (upper-left) for recent decades. While the attribution cannot be made from the observational results alone, we could decompose a forced signal and internal variability in the PC2 using MIROC6 large ensemble simulations (Fig. 3d) based on the fact that the observed multidecadal variability was reproduced by the model well. Our results strongly suggest that the recent PC2 trend arising from the positive AMV trend has been forced externally, consistent with previous studies that show the aerosols driving of the AMV (Booth et al. 2012, Watanabe and Tatebe 2019, Qin et al. 2020).

3. There is no text detailing the code and data availability. Is code going to be made available for reproducibility of results? I am not familiar with if this journal requires this.

This comment was given by Editor, too. We have now added Data and Code Availability sections that meet the condition of Nature Communications.

4. L196: If I understand the plot correctly, the mean has moved from a weakening to strengthening. If this is true, it is definitely worth saying.

Following the suggestion, we have explicitly stated the shift from a weakening to a strengthening after correction at L.182–184.

NCOMMS-25-54711-T

North Atlantic influence reconciling model-observation discrepancy in the tropical Pacific warming pattern

by Lin and Watanabe

Reply to the comments by Reviewer #3

We thank the reviewer for carefully reading our manuscript and providing comments. Please see our point-by-point responses below.

> *The study examines potential sources of error in models for incorrectly simulating multi-decadal trends in the zonal gradient of sea surface temperatures (SSTs) in the tropical Pacific. The authors find a relationship between the zonal tropical Pacific SST gradient and multi-decadal variability in Atlantic SSTs in reanalysis products. The relationship indicates that positive values of Atlantic multi-decadal variability (e.g., positive SST anomalies in the Atlantic) yields a strengthened zonal SST gradient across the tropical Pacific (and presumably, the opposite for negative Atlantic SST anomalies). However, climate model simulations show the opposite relationship - i.e., positive Atlantic SST anomalies in the Atlantic over multiple decades leads to a weaker zonal tropical Pacific SST gradient. The authors use some statistical techniques to show that when the Atlantic multi-decadal SST pattern is corrected in the models, the representation of the tropical Pacific zonal SST gradient is improved. However, no dynamical improvements to the models were offered or considered.*

Thank you for the comment. The primary goal of our study was to identify a previously underappreciated source of the model–observation discrepancy in tropical Pacific SST trends, namely biases in the AMV–Pacific teleconnection in large ensembles of climate models. We do not imply to offer specific approach to improve the models as the above bias might have occurred due to multiple reasons (insufficient resolution, errors in parameterization schemes, etc). The “reconciling” analysis in Figs. 4 and 5 was performed as a statistical “what-if” test without carrying out further numerical experiments: by correcting the AMV-related teleconnection pattern and removing the biased forced component in IPO, we demonstrated that these biases have a non-negligible impact on the simulated ΔSST_x trends and can substantially reduce the discrepancy with observations. Designing and performing targeted sensitivity experiments to verify the above statistical tests would be an important

next step before proposing how to improve models, and the possible ideas have now been explained in the last section.

The study addresses a very timely and important topic in climate dynamics - the poor simulation of tropical Pacific SST variability in climate models, particularly their fidelity in reproducing observed trends in the field. The authors offered an interesting way of examining this bias in a statistical way and have a "proof of concept" with a few models.

Thanks!

However, there are questions as to how generalizable this method is with other models. And certainly, the authors could explore more models from the CMIP6 database to make their point. Even a doubling of the number of models would bolster their case further. Or, in contrast, finding models which actually do simulate the observed trend in the tropical Pacific zonal SST gradient fairly well and contrast those with these models. But, that was not done and leaves a gaping hole in the analysis that is in the scope of the work.

Thank you for the constructive suggestion. In the revised manuscript, we have extended the multi-model analysis by adding five additional CMIP5/CMIP6 models, which provided large ensemble outputs, and doubling of the number of models did increase our confidence about the conclusions. The updated Figs. 5 and 6, together with the individual PDFs in Supplementary Fig.12, shows that the main result from MIROC6 also appears in other models. Also, our revised Fig. 5 (and some previous studies such as Wills et al. 2022 GRL) shows that basically none of CMIP models could properly reproduce the observed ΔSST_x trends, so that we did not take a good/bad model approach.

Another issue I have with the manuscript is that there are WAY TOO MANY Supplemental Figures to consider for the analyses. Supplemental Figures should only be used to further support some arguments in the paper, not be relied on to make actual arguments or support of the hypotheses. However, this is exactly what the authors do - e.g., the "Multi-model analysis" section of the paper refers only to Supplemental Figures (and many of them!) to present results. This over-reliance on Supplemental Figures is a classic signal is that the study is not well-equipped for a short format journal like this. And, with my suggested addition of examining more models and contrasting "poor-performing" versus "better" models, the main sections of the paper

would need to be expanded even further.

We appreciate the reviewer's concern about the number and role of the Supplementary figures. We would first like to clarify that the key results of the multi-model analysis were presented in the main text. In particular, Fig. 5 showed that the reconstruction test applied to the MIROC6 large ensemble can work for other CMIP large-ensemble models. The Supplementary figures relevant to the multi-model analysis, especially EOF patterns and PC time series for individual models, were presented to allow readers to trace the results of model-by-model details, rather than to introduce additional core arguments beyond those discussed in the main text. In response to the comment, we have revised Supplementary Information by combining multi-model EOF and PC figures into two (Supplementary Figs. 8 and 9). With this reconfiguration, the number of Supplementary figures about multi-model analysis has reduced from seven to five in the revised Supplementary Information despite that we have doubled the number of models according to a suggestion by the other Reviewer. Regarding the suggestion to contrast poor and better models, please see our reply to your previous comment above.

For these reasons, I am recommending that the paper be rejected for publication in Nature Communications and instead be resubmitted to another longer-format journal for publication.

While we respectfully disagree with your assessment in our study, we have revised the manuscript to clarify the role of the Supplementary Figures and to ensure that the main conclusions are presented compactly in the main text.

Reviewer #3 (Remarks on code availability):
There was no code available for review.

This comment was given by Editor, too. We have now added Data and Code Availability sections that meet the condition of Nature Communications.

NCOMMS-25-54711-T

North Atlantic influence reconciling model-observation discrepancy in the tropical Pacific warming pattern

by Lin and Watanabe

Reply to the comments by Reviewer #2

We thank the reviewer for the careful second assessment of our manuscript and for the positive overall evaluation. We have revised the manuscript according to your suggestion and comments. Please see our point-to-point reply below.

1 Comments to the editor

The authors have carefully considered my comments, and those of the other reviewers, and made many positive changes to the manuscript.

Under the recommendation of Reviewer 1, the authors have added additional models (Fig 5) and this has strengthened the paper. Reviewer 3 suggested that there were too many SI figures. I agree with this point, but I think there is a nice balance the authors find in mentioning interesting things in the SI without relying on it too much in the paper. I think it is fine to leave it up to the reader to decide if they want to know more. There may have been a missed opportunity to have this in a longer format paper, this is the authors choice and I have no problem with it.

2 Response to my previous review

Thank you for your detailed notes in response. Thanks for adding the PDO sentence to the introduction. The methods section is significantly improved and much easier to follow now.

3 Summary of current review

By expanding the methods section I understand the paper more clearly. The authors have provided a very thorough analysis and I suggest the paper is published with only a few minor suggestions. Please note, that all line numbers below are in reference to the updated manuscript.

Thank you!

4 Minor Comments

1. Please add a reference to support the 1.1K statement on L18.

An appropriate reference has now been added to support this statement: the IPCC AR6 Synthesis Report (IPCC, 2023).

2. Could you expand a little on the point in the introduction about the dependence on the time period? I think the mention is too brief.

The relevant sentence has been expanded to clarify the reason more explicitly, by noting that low-frequency internal variability can affect the tropical Pacific SST trend estimated over a given interval (L.29–31).

3. Fig 4 and 5. These plots are not very colour blind friendly. Suggest you adjust colour palette.

The colour palette in Figs. 4 and 5 has been revised to improve colour-blind accessibility.

4. Please add to the description the significance level selected for stippling on maps.

All relevant figure captions have been revised to state the significance level used for stippling explicitly (95% confidence level).

5 Editing Comments

1. In the methods section, can you make it clearer that the precip and wind data was only used for figures in the SI. As these datasets are not needed for your main paper figures (but agree the refs need to be in the main paper so mention of them is fine).

This has now been clarified in the Methods by explicitly noting that these atmospheric datasets were used only for the supplementary figures (L.540–542).

2. In my opinion, some of the model descriptions within "Large ensemble climate simulations" would be better in the SI. The key thing is that the references remain in the text and perhaps a very short description on the range of model resolutions (which is currently missing).

The model descriptions in "Large ensemble climate simulations" have been streamlined, with additional details moved to Supplementary Text 1. The key references remain in the main text, and a brief description of model resolution has been added.

3. Fig 1. The subplot annotation of a-d feels unnecessarily large (and in other locations too). Suggest rescaling the colour bar to divisions of .1 or similar so only 1 dec place is needed (like in fig 3). These are personal choices and I am happy for the authors to decide if these suggestions are helpful or not.

The figure design was revised accordingly to improve readability, including reducing the size of subplot annotations.

6 Additional Literature to cite
I noticed a couple of papers I would have expected to be cited. Adding them for your consideration
1. Jiang et al (2025): Subsurface cooling and sea surface temperature pattern formation over the equatorial pacific.
2. Simpson et al (2025): Confronting earth system model trends with observations.
3. Dhame (2025): Higher-resolution climate models do not consistently reproduce the observed tropical pacific warming pattern.

Thank you for these helpful suggestions. Jiang et al. (2025) has now been cited in the Introduction, where we discuss possible model biases in the externally forced response related to tropical Pacific SST pattern formation (L.40-41). Dhame et al. (2025) has also been added in the Introduction (L.44), in the context of the broader model–observation discrepancy in the tropical Pacific warming pattern. Simpson et al. (2025) was also considered carefully but was not included because its scope is broader and partly overlaps with the literature already cited. We therefore chose to keep the reference list focused on studies more directly related to the specific mechanisms and diagnostics examined in this manuscript.

NCOMMS-25-54711-T

North Atlantic influence reconciling model-observation discrepancy in the tropical Pacific warming pattern

by Lin and Watanabe

Reply to the comments by Reviewer #3

We thank the reviewer for the careful second assessment of our manuscript and for the constructive feedback on the revised version. We have addressed the remaining comments and revised the manuscript accordingly. Please see our point-by-point responses below.

Overall Opinion

This is my second review of this manuscript. I thank the authors for their detailed responses to the reviewers' comments, including mine. The authors have made a genuine effort to address the concerns raised and make meaningful changes to the paper. Several additions such as the ANOVA decomposition, the sensitivity tests for the detrending methodology, and the expansion of the multi-model analysis to more models (my primary criticism), collectively strengthen the paper's conclusions. My original review noted that the paper seemed unsuitable for the journal not because of the topic or the novelty of the work but mostly because of its overall length. The authors reduced the number of Supplementary Figures, though there are still a lot of them. I won't harp on the issue, as it seems the editor is OK with the number of Supplementary Figures. The paper continues to be of interest and timely for the climate community. I am overall supportive of the research and how it can advance multi-decadal variability and our knowledge of inter-basin teleconnections.

Thank you!

However, some scientific concerns remain that need to be addressed before publication. Therefore, I am recommending that the paper undergo major revisions.

Major Revisions

1. The teleconnection mechanism remains underexplained. The authors state that climate models simulate the wrong sign of the AMV–tropical Pacific SST gradient relationship and demonstrate

statistically that correcting this pattern reduces the model–observation discrepancy. However, the dynamical reason for this teleconnection bias is not adequately addressed. The authors mention that biases in climatological mean states in the tropical Pacific could be a cause, and flux-adjusted pacemaker experiments are mentioned as potential future work. While I understand a full mechanistic explanation is beyond the scope of the paper, at a minimum, the discussion section should mention possible mechanisms – e.g., biases in the Walker Circulation response to North Atlantic heating, known biases in the ITCZ representation in CMIP6 models, or other possibilities the authors think are worthy to mention.

We appreciate the reviewer's helpful suggestion. In response to your comment, we have expanded the discussion in the "Implications and remaining issues" section (L.244–249) to better describe plausible mechanisms underlying the AMV–Pacific teleconnection bias. Specifically, we have mentioned that possible reasons for this model's failure are the tropical Pacific mean-state biases. In particular, a too-weak cold tongue and biased ITCZ may distort the atmospheric response in the tropical Pacific (Seager et al., 2019; Seager et al., 2022; Jiang et al., 2025; Zhuo et al., 2025), thereby weakening or shifting the Walker circulation and cross-equatorial wind anomalies triggered by North Atlantic warming (McGregor et al., 2014; Li et al., 2016; Sun et al., 2017; Sun et al., 2021).

2. Stationary Statistics. In the response to one of the reviewers, the authors state that the ensemble-mean correlation between the observed PC-2 time series and the model ensemble-mean PC-2 time series is r = 0.83. This correlation is used to suggest that potential errors are not from the time evolution of AMV but instead the teleconnection pattern between the AMV and the tropical Pacific. However, what if the teleconnection is not stationary in time? What if the role of external forcing is to change the nature of the connections between the AMV and the tropical Pacific? This possibility could be explored in the models and reanalysis by examining rolling correlations between the AMV index and the teleconnection pattern. If the relationship has changed over time, then the "biases" in the model's simulations of the relationship may not be bias after all. Instead, the limited sample size in observations is skewing our understanding of the relationship. This point is worth exploring and having a short discussion (2 or more sentences) in the manuscript.

We thank the reviewer for the insightful comment. We agree that the model–observation difference

in EOF2 could partly reflect temporal changes in the relationship rather than a model bias if the AMV-related teleconnection were strongly non-stationary. To address this point, we have added a new stationarity analysis in the Methods (*“Stationarity of the AMV-related teleconnection”*) and a new Supplementary Fig. 16. Specifically, we have repeated the SST regression onto PC2 using rolling 44-year windows within 1920–2023 and computed the spatial correlation between each window-specific regression map and the corresponding full-period pattern over the Pacific domain.

In observations, the similarity to the full-period pattern is lower in the earlier windows (e.g., r = 0.44 for 1935–1978) but increases toward recent decades, reaching r = 0.89 for 1979–2022. This indicates that the observed relationship is less coherent in the early record, whereas the late-period pattern most relevant to the recent model–observation discrepancy already closely resembles the full-period structure. In MIROC6, the corresponding correlations remain high throughout the record (r = 0.93, 0.89, 0.98, and 0.95 for 1935–1978, 1951–1994, 1963–2006, and 1979–2022, respectively), indicating that the AMV-related teleconnection pattern is broadly stable in time. We therefore added a short discussion in the main text (L.149–151) noting that the teleconnection bias appears temporally robust in MIROC6. Overall, these results suggest that the model–observation difference primarily reflects a persistent bias in the spatial structure of the AMV-related Pacific response.

> *3. Sample Size for Statistical Significance. The Methods section of the paper mentions that the authors use a two-sided t-test when conducting significance for their regression and correlation analyses. However, there is no mention of how serial correlation is considered for the degrees of freedom. Since indices and several fields in this manuscript has low-frequency variability, there is a significant reduction in the number of independent samples. The authors should state explicitly what they used as their sample size and, if not done, they should correct their degrees of freedom to account for the serial correlation and redo the statistical significance on their plots.*

Thank you for raising this point. Serial correlation has now been accounted for in the statistical significance test using the effective degrees of freedom, following previous studies (Davis, 1976; Zhao and Khalil, 1993). This has been clarified in the Methods (*“Regression and PDF.”*) (L.515–517), and the significance masks in the revised figures were recalculated and updated accordingly.

Reviewer #2
(Remarks to the Author)

Review of 'North Atlantic influence reconciling model-observation discrepancy in the tropical Pacific warming pattern'

by Yueh-Chi Lin and Masahiro Watanabe

Nature Communications manuscript number: NCOMMS-25-54711-T

Recommendation: Major Revision

Reviewer Name: Penelope Maher

1 Summary of the Review

This manuscript investigates the strengthening in the tropical Pacific SST gradient in observations and seeks to explain why climate model simulation have a weaken SST gradient. The authors attribute model biases in the Atlantic multidecadal variability as a key reason to explain why models fail to capture the observed SST strengthening. The authors have carried out an interesting analysis and have generated a large number of figures to support their findings. I note that Professor Watanabe is a world leader in this area of research and, as such, is an authority on this topic.

I anticipate the manuscript will be suitable for publication in Nature communications. I have two main areas which impacted by ability to follow the analysis. While reading the manuscript this became a distraction to my enjoyment of the article, so for this reason I as suggesting major revisions. I noticed quite of lot of small text changes that I could suggest, but I will include these in the second round of review, as I don't want them to distract from the science. Nature communications is a journal with a quick turn around so I don't expect this will delay the process.

2 Major Comments

1. The IPO is assumed to be the prominent multi-decadal influence of the tropical Pacific gradient. The PDO is also known to impact the the gradient as well. Could I suggest you justify why the IPO was considered but not the PDO as well please? I realise the PDO is more constrained to the NH, but it still impacts the tropical Pacific SSTs. Perhaps it is as simple as the IPO and PDO are phase locked but I would like to hear the authors thoughts and justifations on this please.

2. Reconstruction tests: I like the idea and I applaud the effort, but I don't understand what has been done so I am struggling to know if I agree with your assessment or not. In L184-188 the method is described, but not in enough detail so that I know what has been done. Could you comment on if this is a common method (I am not familiar with it), there are no references in this section it might help to add some, and I suggest you expand the methods section to explain this in more detail. Could you also clearly say this is a statistical test of the model data and that simulations were not repeated with altered model set-up. This was my initial thought until I realised you have done it for the other models too.

3 Minor Comments

1. In 2a, the colour scale in the background represents the trends. Could you comment on the anticorrelated nature of the two PCs please? (i.e. why a PC1 of .1 related to a PC2 of -.1). I want to better understand the inherent structure behind the dots.

2. Fig 2. I like it. I took me a while to 'get it' but it is interesting. Do you think we can say anything about the forced response by the fact the shape is not a closed circle (i.e. a lifecyle of a mode of variability) and that the latter 10 time windows or so move into a new part of the phase space?

3. There is no text detailing the code and data availability. Is code going to be made available for reproducibility of results? I am not familier with if this journal requires this.

4. L196: If if I understand the plot correctly, the mean has moved from a weakening to strengthening. If this is true, it is definitely worth saying.

Reviewer #2
(Remarks to the Author)

Review of 'North Atlantic influence reconciling model-observation discrepancy in the tropical Pacific warming pattern'

by Yueh-Chi Lin and Masahiro Watanabe

Nature Communications manuscript number: NCOMMS-25-54711-T

Recommendation: Accept following very minor comments.

Reviewer Name: Penelope Maher

1 Comments to the editor

The authors have carefully considered my comments, and those of the other reviewers, and made many positive changes to the manuscript.

Under the recommendation of Reviewer 1, the authors have added additional models (Fig 5) and this has strengthened the paper.

Reviewer 3 suggested that there were too many SI figures. I agree with this point, but I think there is a nice balance the authors find in mentioning interesting things in the SI without relying on it too much in the paper. I think it is fine to leave it up to the reader to decide if they want to know more. There may have been a missed opportunity to have this in a longer format paper, this is the authors choice and I have no problem with it.

2 Response to my previous review

Thank you for your detailed notes in response. Thanks for adding the PDO sentence to the introduction. The methods section is significantly improved and much easier to follow now.

3 Summary of current review

By expanding the methods section I understand the paper more clearly. The authors have provided a very thorough analysis and I suggest the paper is published with only a few minor suggestions. Please note, that all line numbers below are in reference to the updated manuscript.

4 Minor Comments

1. Please add a reference to support the 1.1K statement on L18.
2. Could you expand a little on the point in the introduction about the dependence on the time period? I think the mention is too brief.
3. Fig 4 and 5. These plots are not very colour blind friendly. Suggest you adjust colour palette.
4. Please add to the description the significance level selected for stippling on maps.

5 Editing Comments

1. In the methods section, can you make it clearer that the precip and wind data was only used for figures in the SI. As these datasets are not needed for your main paper figures (but agree the refs need to be in the main paper so mention of them is fine).
2. In my opinion, some of the model descriptions within "Large ensemble climate simulations" would be better in the SI. The key thing is that the references remain in the text and perhaps a very short description on the range of model resolutions (which is currently missing).
3. Fig 1. The subplot annotation of a-d feels unnecessarily large (and in other locations too). Suggest rescaling the colour bar to divisions of .1 or similar so only 1 dec place is needed (like in fig 3). These are personal choices and I am happy for the authors to decide if these suggestions are helpful or not.

6 Additional Literature to cite

I noticed a couple of papers I would have expected to be cited. Adding them for your consideration

1. Jiang et al (2025): Subsurface cooling and sea surface temperature pattern formation over the equatorial pacific.
2. Simpson et al (2025): Confronting earth system model trends with observations.
3. Dhame (2025): Higher-resolution climate models do not consistently reproduce the observed tropical pacific warming pattern.

Manuscript: NCOMMS-25-54711-T
Title: North Atlantic influence reconciling model-observation discrepancy in the tropical Pacific warming pattern
Authors: Lin and Watanabe

Overall Opinion

This is my second review of this manuscript. I thank the authors for their detailed responses to the reviewers' comments, including mine. The authors have made a genuine effort to address the concerns raised and make meaningful changes to the paper. Several additions such as the ANOVA decomposition, the sensitivity tests for the detrending methodology, and the expansion of the multi-model analysis to more models (my primary criticism), collectively strengthen the paper's conclusions. My original review noted that the paper seemed unsuitable for the journal not because of the topic or the novelty of the work but mostly because of its overall length. The authors reduced the number of Supplementary Figures, though there are still *a lot* of them. I won't harp on the issue, as it seems the editor is OK with the number of Supplementary Figures.

The paper continues to be of interest and timely for the climate community. I am overall supportive of the research and how it can advance multi-decadal variability and our knowledge of inter-basin teleconnections. However, some scientific concerns remain that need to be addressed before publication. Therefore, I am recommending that the paper undergo **major revisions.**

Major Revisions

1. **The teleconnection mechanism remains underexplained.** The authors state that climate models simulate the wrong sign of the AMV–tropical Pacific SST gradient relationship and demonstrate statistically that correcting this pattern reduces the model–observation discrepancy. However, the *dynamical* reason for this teleconnection bias is not adequately addressed. The authors mention that biases in climatological mean states in the tropical Pacific could be a cause, and flux-adjusted pacemaker experiments are mentioned as potential future work. While I understand a full mechanistic explanation is beyond the scope of the paper, at a minimum, the discussion section should mention possible mechanisms – e.g., biases in the Walker Circulation response to North Atlantic heating, known biases in the ITCZ representation in CMIP6 models, or other possibilities the authors think are worthy to mention.
2. **Stationary Statistics.** In the response to one of the reviewers, the authors state that the ensemble-mean correlation between the observed PC-2 time series and the model ensemble-mean PC-2 time series is r = 0.83. This correlation is used to suggest that potential errors are not from the time evolution of AMV but instead the teleconnection pattern between the AMV and the tropical Pacific. However, what if the teleconnection is not stationary in time? What if the role of external forcing is to change the nature of the connections between the AMV and the tropical Pacific? This possibility could be explored in the models and reanalysis by examining rolling correlations between the AMV index and the teleconnection pattern. If the relationship <u>has</u> changed over time, then the "biases" in the model's simulations of the relationship may not be bias after all. Instead, the limited sample size in observations is skewing our understanding of the relationship. This point is worth exploring and having a short discussion (2 or more sentences) in the manuscript.
3. **Sample Size for Statistical Significance.** The Methods section of the paper mentions that the authors use a two-sided t-test when conducting significance for their regression and correlation analyses. However, there is no mention of how serial correlation is considered for the degrees of freedom. Since indices and several fields in this manuscript has low-frequency variability, there is a *significant* reduction in the number of independent samples. The authors should state explicitly what they used as their sample size and, if not done, they should correct their degrees of freedom to account for the serial correlation and redo the statistical significance on their plots.
